# Targeting materials and strategies for RNA delivery

**DOI:** 10.7150/thno.87316

**Published:** 2023-08-21

**Authors:** Lixin Lin, Kexin Su, Qiang Cheng, Shuai Liu

**Affiliations:** 1 College of Pharmaceutical Sciences, Zhejiang University, Hangzhou 310058, China.; 2 Department of Biomedical Engineering, College of Future Technology, Peking University, Beijing 100871, China.; 3 Liangzhu Laboratory, Zhejiang University, Hangzhou 311121, China.; 4 Eye Center, The Second Affiliated Hospital, School of Medicine, Zhejiang University, Hangzhou 310009, Zhejiang, China.; 5 National Key Laboratory of Advanced Drug Delivery and Release Systems, Zhejiang University, Hangzhou 310058, China.

**Keywords:** Targeting materials, Targeting strategies, RNA-based therapeutics, mRNA delivery, Specific organ/tissue tropism

## Abstract

RNA-based therapeutics have shown great promise in various medical applications, including cancers, infectious diseases, and metabolic diseases. The recent success of mRNA vaccines for combating the COVID-19 pandemic has highlighted the medical value of RNA drugs. However, one of the major challenges in realizing the full potential of RNA drugs is to deliver RNA into specific organs and tissues in a targeted manner, which is crucial for achieving therapeutic efficacy, reducing side effects, and enhancing overall treatment efficacy. Numerous attempts have been made to pursue targeting, nonetheless, the lack of clear guideline and commonality elucidation has hindered the clinical translation of RNA drugs. In this review, we outline the mechanisms of action for targeted RNA delivery systems and summarize four key factors that influence the targeting delivery of RNA drugs. These factors include the category of vector materials, chemical structures of vectors, administration routes, and physicochemical properties of RNA vectors, and they all notably contribute to specific organ/tissue tropism. Furthermore, we provide an overview of the main RNA-based drugs that are currently in clinical trials, highlighting their design strategies and tissue tropism applications. This review will aid to understand the principles and mechanisms of targeted delivery systems, accelerating the development of future RNA drugs for different diseases.

## Introduction

The world has recently witnessed the remarkable impact of messenger RNA (mRNA) vaccines in effectively combating the COVID-19 pandemic, opening a new era of RNA drugs for therapeutic applications. mRNA therapeutics have demonstrated immense potential in the fields of protein replacement therapies, vaccines, and gene editing, as it can regulate gene expression and produce specialized proteins 1-3. In addition to mRNA, other RNA molecules such as small interfering RNA (siRNA), microRNA (miRNA), and antisense oligonucleotide (ASO) have also been used to treat diverse diseases, including cancer, rheumatoid arthritis, infection, ischemic stroke, and others 4-7. However, RNA drugs all encounter the critical obstacle of precise delivery to targeted sites of interest.

To achieve the intended therapeutic effects, RNA drugs should be precisely delivered to specific organs, tissues, or even cells. Targeted delivery endows several acknowledged advantages. Firstly, delivering RNA cargoes to diseased cells is the prerequisite to realize treatment. Secondly, targeting enables us to address the issue of off-target effects, which often limits RNA therapy by causing toxicity and affecting drug safety as well as efficacy. Precise delivery aims to enhance the bioavailability of RNA drugs at the target site while minimizing their distribution to non-target tissues. Lastly, targeted delivery can lower the required dosage of RNA for administration, improve the biosafety of drugs, and increase the clinical tolerance 8,9.

Despite the enthusiastic potential of RNA-based drugs for targeted treatment of various diseases, multiple obstacles remain to be addressed to fully realize their clinical applications 4,5. One of the major challenges is the instability of RNA molecules. Unlike traditional drugs, RNA molecules are highly susceptible to degradation by endonucleases and hydrolases in blood or physiological fluids, resulting in a short half-life 6. Additionally, the physicochemical properties of RNA, such as its negative charge, hydrophilicity, and high molecular weight, make it difficult for RNA to cross the cell membranes 10-14. Therefore, functional delivery vectors are required to transport RNA to the desired sites. Numerous work on systemic administration of unmodified RNA molecules have indicated that the pharmacokinetic characteristics are usually unsatisfied 7,8. Although chemically modified RNA molecules are significantly more stable, overcoming intracellular barriers for cell entry and endosomal escape remains challenging 15. Hence, to tackle the limitations of RNA-based drugs, rationally designed vectors are needed for targeted delivery of RNA drugs. The targeting vector material design should satisfy the following characteristics: 1) protecting RNAs from degradation in serum; 2) remaining stable in the blood or body fluids; 3) showing excellent biocompatibility and biodegradability; 4) improving the cellular uptake and endosomal escape; 5) providing RNA drugs with a suitable half-life and low toxicity; 6) preventing non-specific interaction with non-target tissues, achieving selective accumulation at the targeted sites, and mediating precise gene regulation 10,16,17.

To address the limitations of RNA-based drugs in practical use, a variety of non-viral vectors have been employed for therapeutics delivery. Until now, to develop targeting materials and strategies remains the critical challenge for RNA drug clinical translation. Although several targeted delivery systems have arisen, the guideline behind organ/tissue tropism remains unclear 18. In this review, we provide an overview of various RNA drugs and their mechanisms of action in exerting therapeutic effects. Following this, RNA targeting materials and strategies are summarized from four factors: the category of vector materials, chemical structures of vectors, administration routes, and physicochemical properties of RNA vectors. Ultimately, we highlight the recent advancements in the clinical applications of RNA-based drugs, particularly mRNA drugs, with a focus on the utility of targeted delivery approaches.

## Mechanisms and therapeutic applications of representative RNA drugs

Different RNA drugs demonstrate distinct characteristics and therapeutic mechanisms 19, and these understandings facilitate to achieve optimal therapeutic effects and appropriate clinical applications. Herein, we summarize the therapeutic mechanisms of several representative RNA drugs and their commonly applied therapeutic fields.

### Messenger RNA (mRNA) drugs

mRNA is a single-stranded ribonucleotide transcribed from a template strand of DNA, which carries the genetic code and directs the synthesis of proteins. Natural mRNA is composed of the following parts: 5' and 3' untranslated regions (UTRs), 3' poly (A) tail, 5' cap. Among these, UTRs do not encode proteins, but can regulate locations of mRNA and translation efficiency 9,20. The 5'cap, also known as m^7^GpppN, can bind to the multi-subunit initiation factor eIF4F to promote mRNA binding with ribosomes and translation of mRNA 21. Simultaneously, this cap structure can protect mRNA from degradation by exonuclease 10. The length of poly A is generally 30-70 nucleotides, which is known to influence the half-life and translation efficiency of mRNA, and it is reported that 120 nucleotides can enhance the stability of mRNA 11,12.

Compared to DNA drugs, mRNA does not require entry into the nucleus and can function directly in the cytoplasm. This advantage avoids the risk of gene insertion. Additionally, mRNA has transient expression and will be degraded by enzymes in a limited time after entering the body. This property endows mRNA high safety for therapeutic use 13. After *in vitro* transcribed (IVT) mRNA is delivered into the desired cells by a suitable vector, such as lipid nanoparticle (LNP), the mRNA is released from the vector. Then the mRNA directs protein synthesis with the help of ribosomes and transfer RNA (tRNA), which can be used for protein replacement to treat protein deficiency (e.g. haemophilia B) or protein malfunction (e.g. muscular dystrophy) 14,22.

To date, one of the most significant applications of mRNA is to be used as the vaccines in the field of cancer immunotherapy and viral infections. The immune system plays a crucial role in fighting cancer and protecting the body from bacteria invasion. However, tumor-associated cells can secrete a variety of cytokines and chemokines through different mechanisms to inhibit immune system activities 23. Individuals with weakened or inactive immune systems are more vulnerable to viruses and bacteria. mRNA can be utilized as vaccines by encoding the antigens of tumors or viruses and incorporating them into suitable vectors 24. Upon appropriate administration, mRNA is translated into proteins, which are then degraded by the protease into small fragments, known as antigenic peptide epitopes. Afterwards, the fragments will be taken up by MHC molecules and immune response will be enhanced to activate CD4^+^ and CD8^+^ T cells and B cells, killing and eliminating viruses or tumor cells 15. For instance, Oberli *et al.* used LNPs to deliver mRNA encoding melanoma-related antigens, which induced a strong immune response and prolonged the survival time of mice bearing melanoma 16. However, no RNA-based drugs for treating cancer have been approved by FDA thus far. In this case, the marketing of two mRNA-based COVID-19 vaccines is encouraging. Compared to the inactivated viral vaccine, the mRNA vaccines have shown the ability to elicit a stronger immune response, resulting in higher antibody level and higher protection rate of over 90% 25,26. To cope with the virus's tendency to mutate, it is crucial to design vaccines that are effective against of viruses in different periods. One notable advantage of mRNA vaccines is that they can be rapidly designed and mass-produced, tailoring the characteristics of different virus strains. For instance, Moderna took only 63 days to inoculate the first dose of mRNA vaccine after completing of gene sequencing 19, which was also benefited from the advancement of LNP delivery technology. The success of mRNA vaccine has drawn increased attention to the potential of RNA-based drugs, simultaneously emphasizing the significance of delivery vector design.

Gene editing is another important therapeutic field of mRNA-based drugs, leveraging the development of Clustered Regularly Interspaced Short Palindromic Repeat (CRISPR)) technology 1. Charpentier and Doudna have been awarded the 2020 Nobel Prize in Chemistry because of their pioneering work on CRISPR-associated protein 9 (Cas9) gene editing technology, proving the significant status of this technology in the field of gene therapy 27,28. The CRISPR-Cas9 system contains Cas9 protein to cleave the genomic DNA and single-guide RNA (sgRNA) to decide the specific location for cutting 29. Considering that to deliver plasmid DNA with Cas9 gene and sgRNA may result in off-target effects with sustaining expression of Cas9 protein and requiring nuclear entry of DNA, in contrast, using IVT mRNA encoding Cas9 protein with the characteristics of transient expression and cytoplasmic functioning can overcome these challenges 1,13,30. Several studies have demonstrated that co-delivering Cas9 mRNA and sgRNA can achieve effective gene editing 17,31-33. Overall, mRNA-based drugs hold tremendous potential in various therapeutic fields and warrant further exploration.

### Small interfering RNA (siRNA) drugs

siRNA, also known as short interfering RNA or silencing RNA, is a type of short double-stranded RNA with the length of 21-23 nucleotides containing 2 nucleotides overhang 34. Once exogenous siRNA enters the cell, it can combine with ribonucleoprotein to form an RNA-induced silencing complex (RISC) and two RNA strands are separated. The remaining antisense (guided) strand is complementary to the targeted mRNA sequence, allowing to guide the Argonaute protein to cut the targeted mRNA, thereby inhibiting the translational process of protein 35. Although siRNA is usually double-stranded, only the antisense strand remains associated with RISC, as the sense strand is rapidly cleared after unwinding 36. The discovery of siRNA earned Fire and Mello the Nobel Prize in Physiology or Medicine in 2006 37, highlighting the significant value and potential of siRNA in medical field. In 2018, the first siRNA drug, Onpattro (Patisiran), was approved by FDA for the treatment of hereditary transthyretin (TTR)-mediated amyloidosis 38. Subsequently, in November 2019, another siRNA drug, Givosiran (Givlaar), was approved by the FDA to treat acute hepatic porphyrias (AHP) 39. Until now, five siRNA drugs have been approved by the FDA, including Patisiran, Givosiran, Lumasiran, Inclisiran, and Vutrisiran, all of which are developed by Alnylam, a pioneering company in the field of RNA therapeutics.

### MicroRNA (miRNA) drugs

miRNA is a single-stranded non-coding RNA molecule that plays a crucial role in the regulation of gene expression and is involved in a series of essential cellular processes, such as cell proliferation, differentiation and apoptosis 40. Similar to siRNA, miRNA can also mediate gene silencing by forming RISC to bind mRNA and inhibit the expression of targeted protein 41. Nowadays, some miRNAs are also discovered to activate the transcription of targeted mRNAs to increase gene expression 44. And this discovery has opened new possibilities for miRNA-based therapeutic strategies. However, no miRNA-based drug has been approved by FDA yet, possibly due to the fact that each miRNA can have hundreds of targets, This phenomenon is known as “too many targets for miRNA effect” (TMTME) 43, making it challenging to achieve specific gene regulation with miRNA, which could lead to unpredictable side effects. Currently, most clinical trials on miRNA drugs have been terminated due to safety issues, with only a few ongoing. Despite these problems, laboratory research continues to uncover the potential of miRNA for the treatment of various diseases such as ovarian cancer, polyomaviruses, and breast cancers 44-46.

### Antisense oligonucleotides (ASOs) drugs

In 1978, Zamecnik and Stephenson reported the use of synthetic oligodeoxynucleotides to inhibit the replication of virus *in vitro*, demonstrating the antisense function of synthetic oligodeoxynucleotides for the first time 47. ASO is a kind of synthesized oligonucleotide with 12-30 nucleotides in length which can bind to targeted RNA sequences through complementary base pairing. This binding can result in RNA cleavage or degradation by RNase H or inhibition of translation through occupancy-only mechanisms 48,49. ASOs have evolved over three generations with different modifications, such as phosphorothioate, alkyl moieties or nucleobase, which can improve stability, prolong circulation time, increase affinity to the target, and reduce off-target effects 49,50. Notably, some specific modifications might confer unique functions. For instance, unlike other RNA-based drugs, most of ASOs do not require a delivery vector, instead, the delivery can be achieved by particular chemical modifications, such as gapmers 51. Encouragingly, numerous candidates have entered clinical trials for the treatment of various diseases, ranging from cancer, infectious disease, neurological disease to metabolic disease, making them the largest class of FDA approved RNA drugs with promising therapeutic potential 50,52,53.

The abundance of RNA species and functions endows huge potential in biological and medical field. However, the efficacy of RNA-based drugs can be affected by the challenge of delivering them to specific organs and sites *in vivo*. To fully realize the potential of RNA-based drugs for disease treatment, further research and strategies are in urgent demand to overcome the problems associated with targeted delivery.

## Materials and strategies to achieve targeting

**Figure 1:**
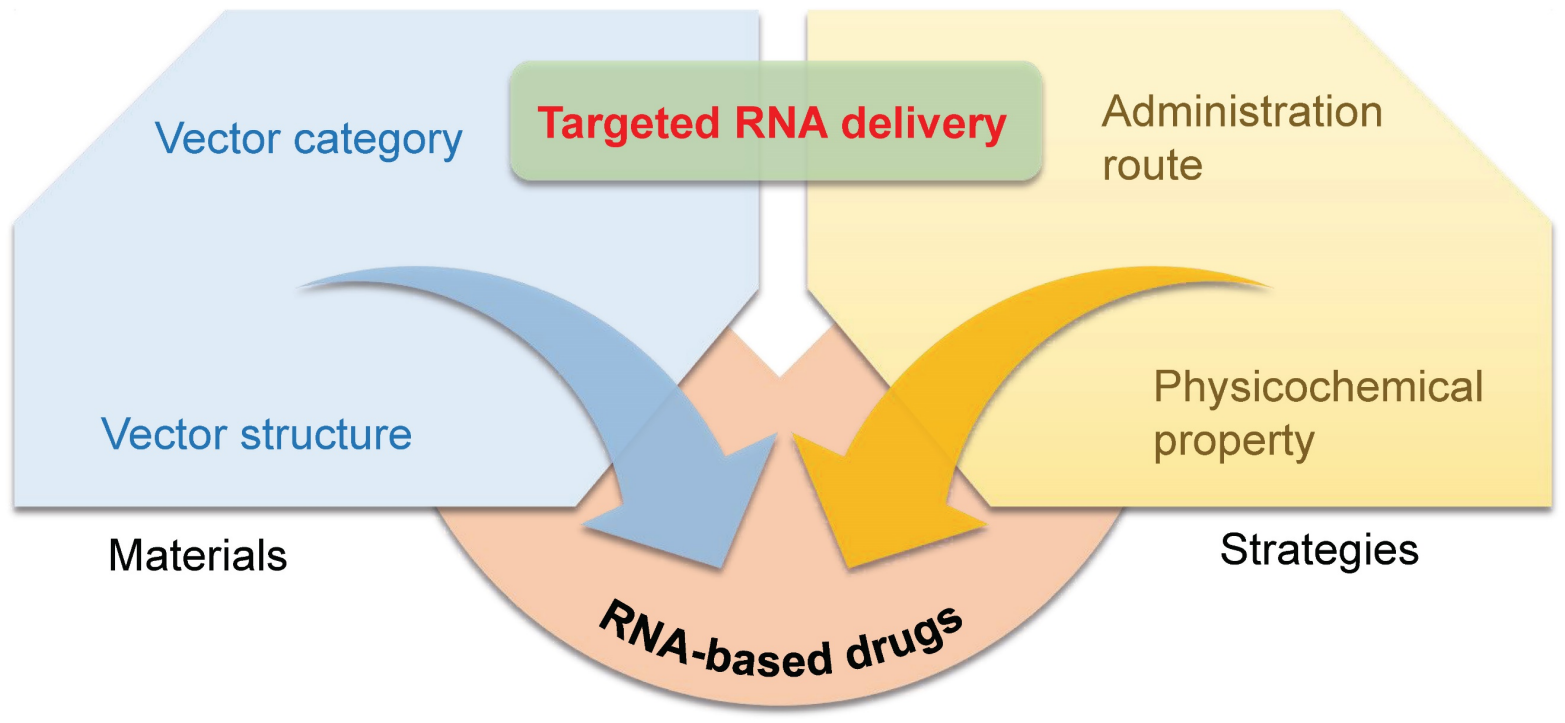
Materials and strategies for targeted RNA delivery. The vector category, vector chemical structure, administration route, and physicochemical property all affect the targeting tropism of nanoparticles.

RNA drugs open an era for treating chronic diseases, nonetheless, their full realization is seriously hindered by lack of targeted delivery technology. Despite the fact that RNA delivery systems have been studied for decades, the precise gene regulation in specific tissues or cells remains a huge challenging. Furthermore, understanding the guideline of organ/tissue tropism of nanoparticles will further the advance of next-generation gene delivery technology, facilitating future RNA-based drug development. Herein, we summarize current progress in targeted RNA delivery, from the point of four key factors: the category of vector materials, chemical structures of vectors, administration routes, and physicochemical properties of RNA vectors (Figure 1).

### The category of vector materials

#### Lipid Nanoparticles (LNPs)

LNPs are one of the widely utilized non-viral vectors for delivering RNA-based drugs. Typically, LNPs are composed of four components with distinct functions, including permanently charged cationic or ionizable cationic lipids, phospholipids, cholesterol and polyethylene glycol (PEG)-lipids 1. Initial LNPs employ permanent cationic lipids, which enable efficient encapsulation of negatively charged RNAs through electrostatic interaction. However, these permanently charged cationic lipids can be toxic to cells and make stimulus after entering the blood, with positive charges, leading to elimination from the body by the immune system 54. Alternatively, ionizable cationic lipids remain neutral in physiological environment, ensuring higher safety, and become positively charged in the acidic endosomal environment of lower pH to facilitate endosomal escape of encapsulated drugs 55. And Lee *et al.* found that ionizable cationic lipids with unsaturated tails could improve the delivery efficiency of LNPs due to the increased lipid fusion ability 56. Cholesterol shows a rigid hydrophobic structure and can be inserted into the gap of the liposome membrane to enhance the stability of the LNP vector. Common helper lipids, such as DOPE, have a relatively small head group to form a tapered shape that facilitates the formation of hexagonal phase II, a transitional form during membrane fusion or bilayer rupture, finally promoting endosomal escape 57,58. PEG-lipids play a significant role in reducing the opsonization effect of serum proteins and non-specific uptake of LNPs, extending the circulation time and half-life of LNPs *in vivo* 59. Recently, studies demonstrated that some LNPs with dual-component enabled mRNA delivery as well 60. Additionally, stability is an important issue to be taken into consideration during storage and transportation of LNP formulations. And changing formulation to lyophilized form or adding cryoprotectant, such as mannitol and sucrose, are feasible methods to improve the stability of LNP formulations 61.

Despite some studies show that lipid materials may cause liver or lung injuries and the cytotoxicity of LNPs is related to the dosage of administration and the properties of lipids used, LNPs offer several advantages compared to viral vectors, including ease of production, relatively low immunogenicity, high RNA loading capacity, and flexible design options 61,62. Most LNPs are prone to target liver and usually be utilized to treat liver diseases. For instance, Onpattro with DLin-MC3-DMA LNP as the vector, is administrated intravenously and enables the inhibition of hepatic production of transthyretin 63. Yang *et al.* demonstrated that LNPs encapsulating HNF4A mRNA could attenuate liver fibrosis in preclinical mouse model 64. Finn *et al.* used a degradable lipid to formulate LNPs to deliver Cas9 mRNA/sgRNA for gene editing *in vivo*. The results showed that the editing efficiency was highly considerable for transthyretin gene editing in the liver, reducing serum protein levels by over 97% with a single dose 65. Recently, some studies have revealed the rules of LNP targeting different liver cell subsets. It is reported that altering surface charge on the LNP in Onpattro formulation from neutral to anionic, the vector was prone to deliver mRNA to the hepatic reticuloendothelial system 66. Additionally, researchers discovered that altering the size, adjusting PEG-lipid content, or incorporating ligands in LNPs affected the distribution of loaded cargoes in hepatocytes and liver sinusoidal endothelial cells (LSECs), or increased the delivery efficiency in extrahepatic organ. For instance, LNPs with 100 nm diameter in size are the most effective for targeting LSECs, while LNPs with smaller diameters, such as 60 nm, showed tropism for hepatocytes 67. Another study showed that incorporating mannose PEG into LNPs led to increased delivery efficiency of siRNA to lungs in mouse model with pulmonary fibrosis after intratracheal injection 68.

#### Polymers

Despite the great potential in clinical translation, extrahepatic RNA delivery by LNPs remains challenging. Alternatively, polymers provide an option for delivery out of the liver. Polymers are divided into different classifications, such as cationic polymers, zwitterionic polymers, polymeric micelles, and dendrimers 1,2,69,70. Polyethyleneimine (PEI) was the “golden standard” for gene delivery previously. The amine-containing PEI can not only improve the membrane affinity of polymeric nanoparticles, for enhanced cellular uptake, but also arise endosomatic effect. The polymers with amine groups show the ability for protonation and leading to osmotic pressure alternation in an acidic endosomal environment, leading to endosome rupture and drug release into the cytoplasm, known as 'proton sponge effect' 71. However, cationic polymers bearing high positive charges would bind to serum proteins and red blood cells, resulting in disruption of plasma membrane 72. Therefore, their applications are severely limited by safety concerns. It was reported that PEI polymers with low molecular weight was less cytotoxic compared to high molecular weight PEI, but the transfection efficacy was also decreased 73,74. Researchers continue to develop next-generation cationic polymers, such as poly(β-amino ester) (PBAE) polymers, which are proved to be less cytotoxic and more efficient than PEI 75-78. Zwitterionic biomaterials with oppositely charged groups have drawn great attention in many fields, like drug delivery, diagnosis, biosensors, and coating. For drug delivery, zwitterionic polymers enable protection of RNA drugs from degradation by inhibiting protein adsorption to extend circulation time. And researches revealed that zwitterionic polymers showed the ability to resist mucus tracking and increase the penetration of mucus to achieve drug delivery 79. In regard to polymeric micelles, Langridge and Gemeinhart demonstrated that polymeric micelles composed of poly(ethylene glycol-block-caprolactone) (mPEG-CL) and poly(ethylene glycol-block-lactide) (mPEG-LA) were stable in cerebrospinal fluid 80, reminding us the potential utility of micelle vectors in targeted cerebrospinal fluid administration. And the variable sizes of polymeric micelles provide the suitable property to penetrate tumor tissue for delivery aim and many laboratory studies verified polymeric micelles could be used to deliver RNA drugs for multiple types of tumors, such as resistant ovarian tumor and solid tumors 81-83. Natural polymers, like chitosan and protamine may overcome the disadvantage of cytotoxicity of synthetic polymers mentioned above, exhibiting good biocompatibility. However, production control between different batches is significant for clinical translation and little difference in molecular weight might alter the delivery efficiency of vectors, which is one main limitation for polymer application in delivering RNA drugs 84.

To further improve the functionalities of vectors, numerous hybrid copolymers, such as lipid-polymer hybrid nanoparticles (LPNs), graphene oxide (GO)-cationic PEI polymers (GO-PEI complexes), polymer-dendrimer hybrids, were developed 1,85. The hybridized polymer-dendrimers exhibited enhanced permeability and retention (EPR) effect, outstanding permeability, and high drug loading capacity. However, several drawbacks still remained: the steric effects of multiple branches in dendrimers might hinder them from degradation, and the excess positive charges could lead lysis of cells and cause cytotoxicity 2,70,85. Conjugation of ligands to improve the targeting ability, masking or reducing charges, and modification of ester bonds to improve degradability might be beneficial for solving these problems.

Numerous novel polymers have been reported to deliver therapeutic cargoes to special organs, particularly the lungs. For instance, hyperbranched PBAEs were synthesized to enable mRNA delivery to the lung epithelium through inhalation, resulting in consistent and controlled protein production without causing pulmonary or systemic toxicity 86. Haque *et al.* utilized chitosan-coated PLGA nanoparticles to encapsulate chemically-modified mRNA encoding human cystic fibrosis transmembrane conductance regulator (CFTR), which was efficiently delivered to lungs of CFTR deficient mice following intravenous and intratracheal administrations 87. Recently, some studies have revealed the potential of polymers to target organs other than the lungs. Liu *et al.* modified cationic polymers by zwitterionic phospholipidation to selectively deliver mRNA to the spleen and lymph nodes after intravenous administration *in vivo*, showing great potential in immunotherapy application 88. McKinlay *et al.* designed charge-altering releasable transporters (CARTs) *via* organocatalytic ring-opening polymerization of various lipids and results demonstrated mixed-lipid CARTs could achieve effective mRNA delivery to B cells and T cells of spleen *in vivo*, and the delivery efficiency was higher than single-lipid CART 89.

#### Exosomes

Exosomes are extracellular vesicles (EVs) with diameters between 30 and 100 nm, and are naturally secreted by cells 90. They play a crucial role in communications between cells *via* ligands, intercellular adhesion molecules on membranes or encapsulated cargo inside the exosomes 91. Compared to other synthesized drug vectors, exosomes show a variety of advantages as natural vectors. For instance, the endogenous origin and membrane proteins of exosomes give them a long half-life in the body 92. Studies have shown that the exosomes derived from foreskin fibroblast of normal human can reduce the phagocytosis of monocytes and macrophages, while enhancing the uptake of cancer cells by micropinocytosis 93. Cancer cells are known to produce a large number of exosomes, and these exosomes show tropism to their source cells, which makes them a potential tool to target cancer cells. For instance, researchers discovered that compared to epithelial cell-derived exosomes, exosomes derived from an ovarian cancer cell line SKOV3 selectively targeted SKOV3 xenograft mice and achieved higher accumulation in tumor site, allowing for CRISPR/Cas9 gene editing to induce the apoptosis of ovarian cancer cells 94. In addition, it was reported that exogenous miRNA-155 mimics or inhibitors were delivered *via* B cell-derived exosomes to hepatocytes or macrophages, with higher delivery efficiency and lower cytotoxicity in contrast to regular transfection methods 95. Specifically, studies showed that the exosomes could cross the brain-blood barrier (BBB), bearing the innate character for brain-targeting 96,97. Perets *et al.* developed a technique to track the exosomes from mesenchymal stem cells of bone marrow (MSC-exo), which could accumulate in mouse brains in different pathological models, including Alzheimer's disease, autism, stroke, and Parkinson's disease. To be more specific, the homing mechanism was driven by inflammation in pathological brains and MSC-exo could be selectively taken up by neuronal cells 98. These findings highlight the potential of exosomes as vector to achieve targeted delivery of RNA-based drugs in various brain pathologic therapies.

#### Inorganic nanoparticles

Inorganic nanoparticles (INPs) are synthesized from inorganic particles, biodegradable polycations, and typical inorganic materials such as gold, silica, metallic oxide, and others. Among INPs, gold nanoparticles (AuNPs) have been extensively studied in inorganic chemistry and nanomaterials 99. AuNPs are chemically inert, showing monodisperse nanostructures without toxicity, making them ideal for functionalization with a variety of ligands 100. One notable application of AuNPs is their interaction with B lymphocytes, enabling targeted immune cell delivery and enhanced immune response. For instance, polymer-coated AuNPs loaded with antigen were utilized to target B lymphocytes and activate CD4 T cell responses 101, which might be used to improve efficacy of vaccines in the future. However, due to the inert property, it is difficult to metabolize AuNPs in time, leading to the long half-life that limits the clinical application of AuNPs. Wang *et al.* utilized copper sulfide, which was metabolizable in liver to improve the excretion of Au. And the results showed that the conjugation of copper sulfide and Au could accelerate the excretion of AuNPs in hepatocytes 102. Other inorganic nanoparticles, such as silica nanoparticles, iron oxide nanoparticles, are also being investigated for targeted applications 99.

### Chemical Structures of vectors

**Figure 2:**
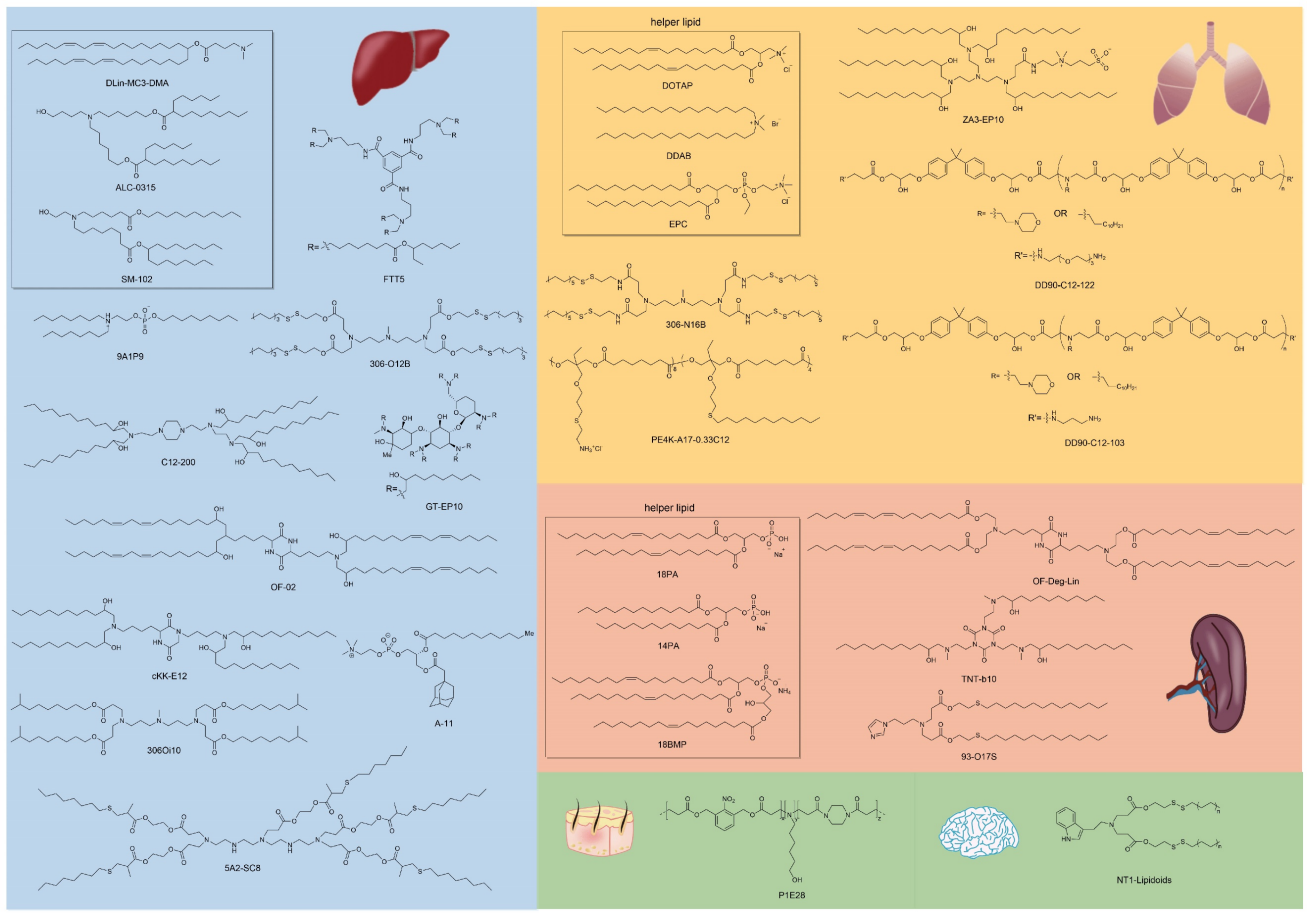
Representative chemical structures of RNA vectors that mediate targeted delivery. RNA vectors for liver, lung, spleen, skin, and brain-targeting are shown.

Similar classes of RNA vectors probably show the same tropism *in vivo*, for instance, most LNPs tend to deliver mRNA into the liver. Nonetheless, minimal alteration of chemical structures of vectors in the same class might also achieve the goal of targeting, and the research on exploring targeted material structures is in full swing. Beyond Dlin-MC3-DMA, ALC-0315, and SM-102 approved by FDA for clinical use in LNP formulations, numerous advancements have been made in the development of various lipids, lipidoids, and polymers for delivering RNA to targeted organs. Here, we outline the relationship between the chemical structures of these materials and their specific targeting capabilities for different organs (Figure 2).

#### Targeting of the liver

**Figure 3:**
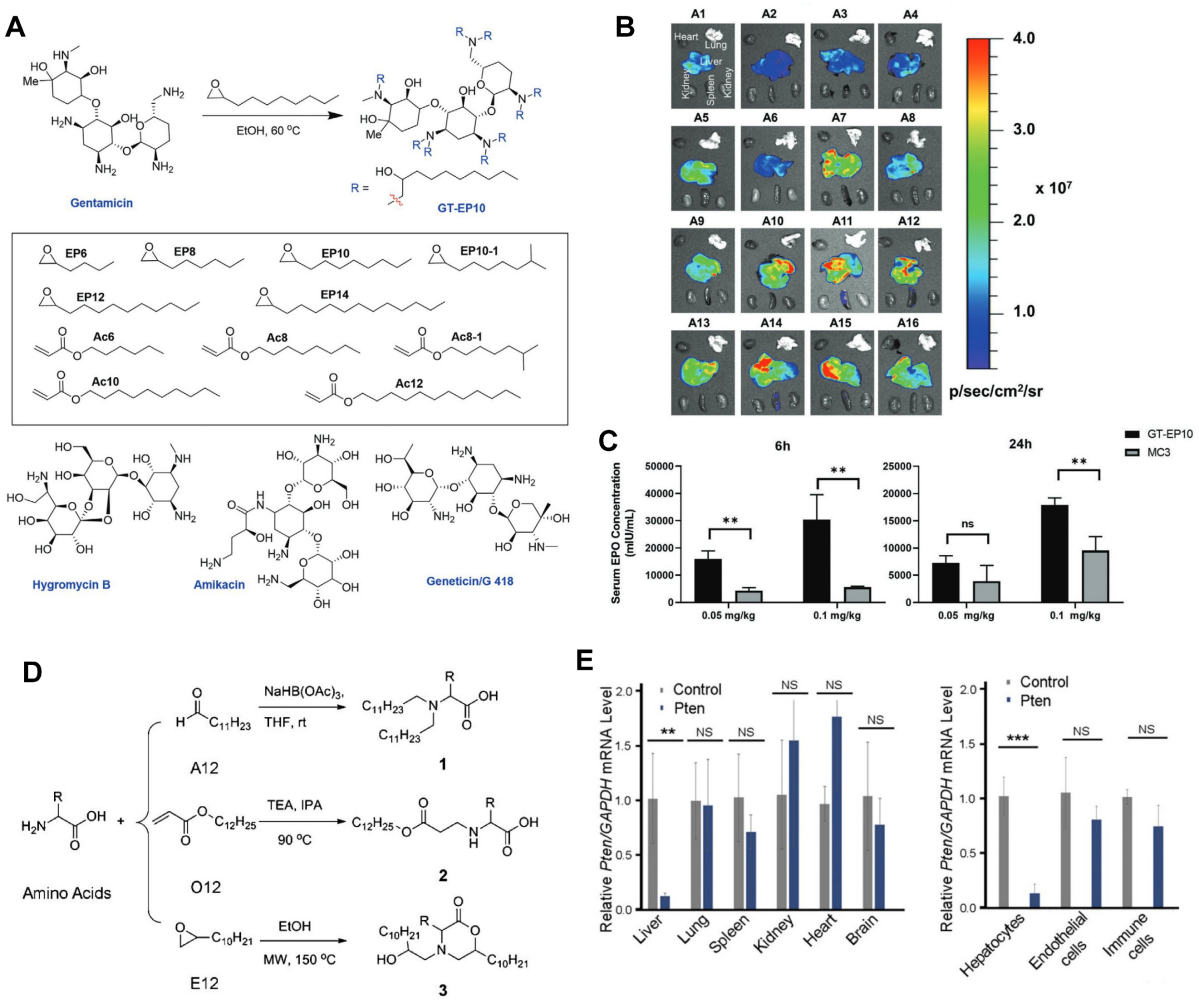
Lipids derived from ring-opening reaction or addition reaction of amines, acrylates, and epoxides for liver-targeting. (A) Synthesis route of cationic lipid-modified aminoglycosides according to ring-opening reaction and structures of aminoglycosides. (B) Luciferase expression *in vivo* of C57BL/6 mice with the delivery of CLA-based LNPs at 6h after intravenous injection. (C) Human erythropoietin expression after 6 h and 24 h with GT-EP10 LNP and MC3 LNP delivery. Adapted with permission from 106, copyright 2020, Wiley-VCH. (D) Synthesis routes of amino acid derivatives by addition reactions. (E) Expression level of Pten, in different organs and subtypes of liver cells. Adapted with permission from 107, copyright 2014, National Academy of Sciences.

Most LNPs tend to target liver, and modifying the LNP vector can enhance their targeting ability or achieve targeting of specific subtypes of liver cells 103-105. For instance, Yu *et al.* developed a class of LNPs with cationic lipid-modified aminoglycosides as shown in Figure 3A, and the results demonstrated that the top-performing GT-EP10 LNPs could deliver FLuc mRNA and human erythropoietin mRNA to liver at a higher delivery efficiency compared to DLin-MC3-DMA LNPs (Figure 3B-C) 106. Gan *et al.* designed a series of LNPs with adamantyl-phospholipids to encapsulate Cre mRNA and DNA barcodes, and intravenous injection allowed the occurrence of tdTomato fluorescence in the liver of Ai14 mice. Further quantification demonstrated that A-11 without specific ligands tended to deliver inclusions to the liver immune cells, instead of common hepatocytes 104. For siRNA delivery, Dong *et al.* designed a library of lipids according to the chemical reaction of amino acids and epoxide or acrylate esters presented in Figure 3D, and identified the top-performing lipid, cKK-E12, *via* iterative screening and structure-activity relationships (SAR) study. As a result, cKK-E12 LNPs delivered siRNA to liver and silenced Phosphatase and tensin homolog (Pten) in hepatocytes with high selectivity (Figure 3E) 107. Furthermore, cKK-E12 LNPs were also utilized to co-deliver Cas9 mRNA and sgRNA for editing PCSK9 in hepatocytes of mice in a subsequent study 103. Similarly, Whitehead and colleagues synthesized a series of lipidoids by Michael Addition for siRNA delivery. And subsequent investigation revealed that the lead 306O_i10_ LNPs resulted in much higher siRNA accumulation in liver than naked siRNA 108.

**Figure 4:**
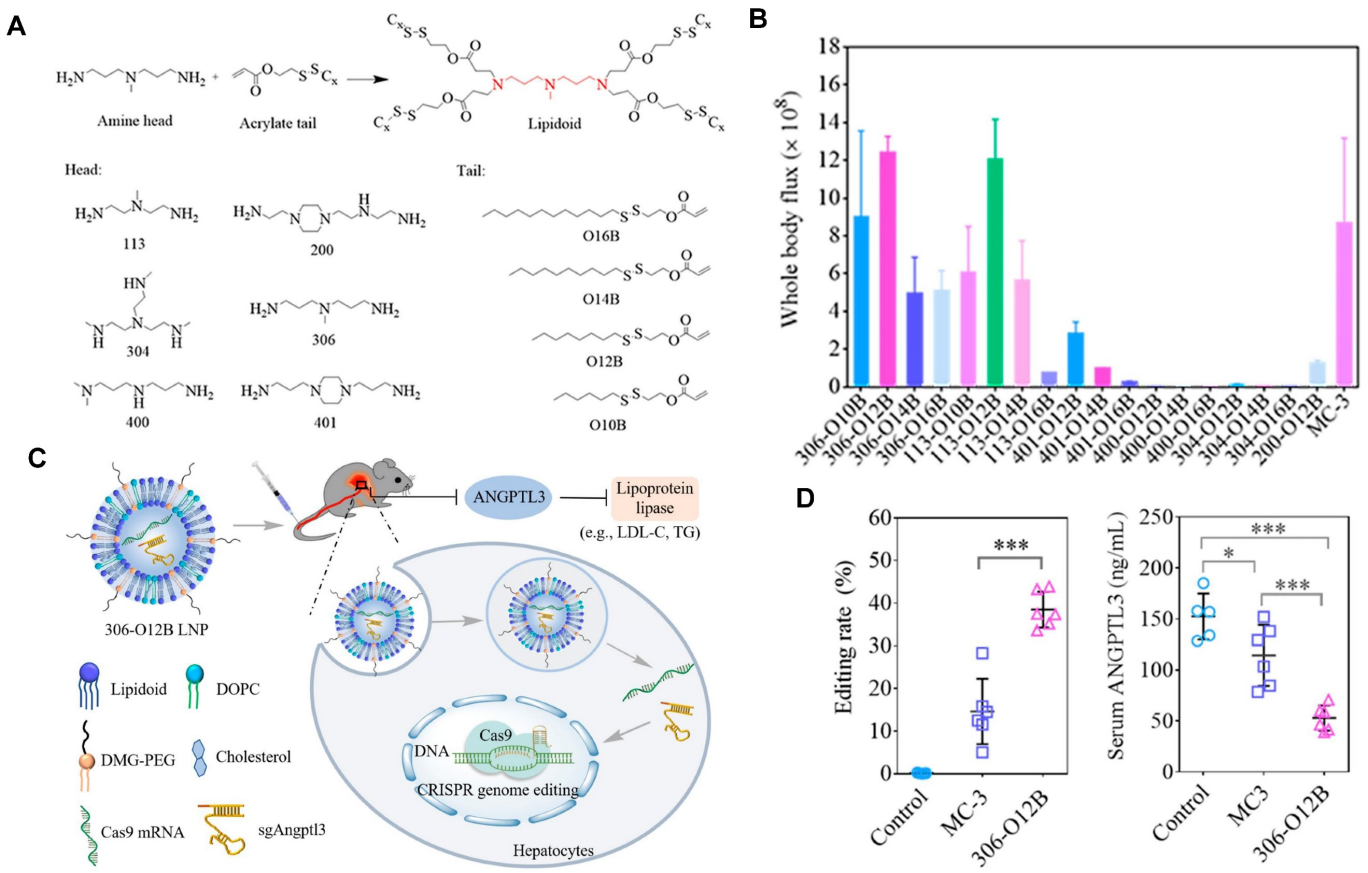
O-series LNPs for liver-targeting. (A) Synthesis of O-series lipidoids. (B) Whole body luminescence intensity of O-series LNPs compared to MC3 LNP in Balb/c mice at 6h after intravenous injection. (C) Schematic illustration of LNP-mediated gene editing in hepatocytes and reduction of *Angptl3* protein resulting disinhibition of lipoprotein lipase. (D) Comparison of *Angptl3* gene editing efficiency with the delivery of 306-O12B LNP and DLin-MC3-DMA LNP. Adapted with permission from 114, copyright 2021, National Academy of Sciences.

Currently, LNPs have been increasingly employed for loading mRNA to achieve liver targeting, and applications such as gene editing and protein replacement are explored 109,110. The mechanism underlying the liver-targeting of LNPs has been extensively studied and several findings are publicized. Upon entry into the body, LNPs can interact with proteins in the biological environment and adsorb proteins on the membrane surface to form the protein corona 55,111. Thus far, it has been demonstrated that the liver-targeting of LNPs is mediated by the absorbed corona proteins, particularly apolipoprotein E (ApoE), which can bind to low-density lipoprotein receptor (LDLR) expressed on hepatocytes and facilitate the uptake of LNP by liver cells 112,113. In a recent study, Qiu *et al.* designed a library of O-series LNPs that contained ester bonds in their tails (Figure 4A). They discovered that 306-O12B LNPs tended to deliver Cas9 mRNA and sgRNA to the liver and led to higher *Angptl3* gene knockdown compared to DLin-MC3-DMA LNPs (Figure 4B-D). Further studies revealed that the differentiation of tissue targeting might be determined by the corona proteins on the nanoparticle surface 114,115.

**Figure 5:**
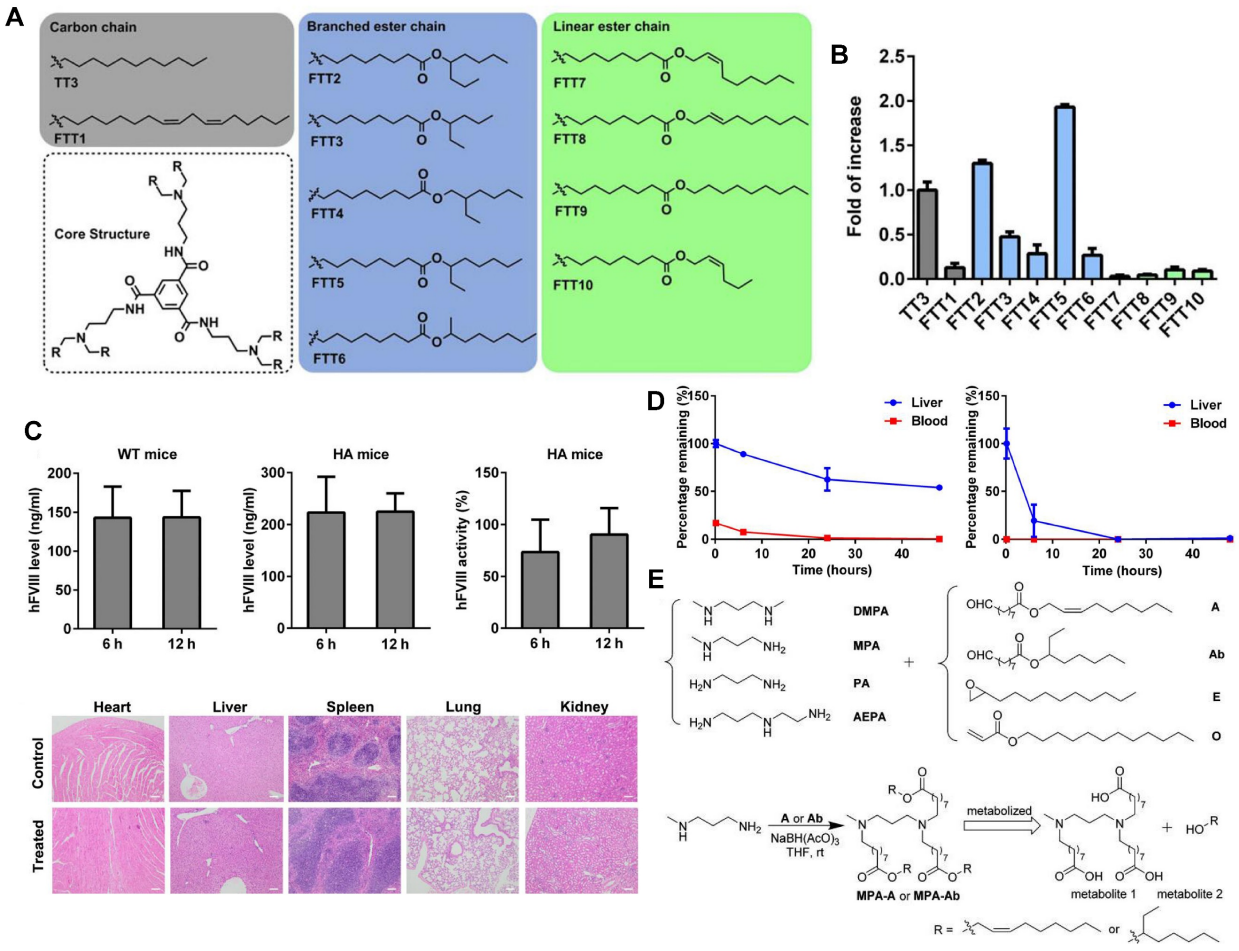
Novel LLNs for liver-targeting. (A) Chemical structures of FTT derivatives with TT3 as core. (B) mRNA delivery efficiency of FTT LLNs was represented by the fold of increase of luminescence intensity *in vivo* and FTT5 showed the highest delivery efficiency. (C) Expression level of hFVIII protein and hFVIII activity in wild-type mice and HA mice after intravenous injection of FTT5-hFVIII mRNA LLNs. And histopathological images of HA mice with injection of FTT5-hFVIII mRNA LLNs and untreated HA mice were shown. (D) FTT5 LLNs with branched ester side chains (left) were less likely to degrade than FTT9 LLNs with linear chains (right) in liver. Adapted with permission from 116, copyright 2020, American Association for the Advancement of Science. (E) The synthesized routes of amino-ester-derived LLNs. Adapted with permission from 117, copyright 2017, American Chemical Society.

Lipid-like nanoparticles (LLNs) represent one of the predominant LNP delivery systems for RNA-based drugs. Dong and colleagues designed a series of LLNs with TT3 as the core structure as depicted in Figure 5A. FTT5 LLNs were formulated to deliver FLuc mRNA *in vivo*, mediating the highest luminescence signal in the liver (Figure 5B). Subsequently, FTT5 LLNs were utilized to package hFVIII mRNA and adenine base editor mRNA, achieving effective hFVIII protein expression and base editing *in vivo* of hemophilia A (HA) mice (Figure 5C). Further study showed that FTT5 LLNs with branched ester side chains exhibited greater resistance to degradation compared to FTT9 LLNs with linear chains (Figure 5D) 116. In an effort to develop biodegradable materials, Zhang *et al.* rationally designed a series of LLNs with different amines and esters, bearing varied carbon chains as shown in Figure 5E. MPA-A and MPA-Ab showed higher Cas9 mRNA delivery efficiency compared to epoxide or acrylate series LLNs and C12-200 LNPs 117. In a subsequent research, Luo *et al.* verified that the optimized MPA-Ab LLN formulations post orthogonal design mediated enhanced mRNA delivery compared to TT3 LLNs 118.

In addition to LNPs mentioned above, several other vectors with different chemical structures can also realize liver-targeting. Rui *et al.* used linear diacrylate as the backbone and monomer E63 with two secondary amines as the end-cap to synthesize PBAE polymers, achieving preferential expression of mRNA in liver 119. In another study, poly(acrylic acid) (PAA8k) was used as the core structure to synthesize polymers by conjugating with oligoalkylamine. The results demonstrated that PAA8k-(2-3-2) encapsulating FLuc mRNA could achieve higher delivery efficiency in the liver compared to PAA8k-(2-2-2) and PAA8k-(3-3-3). Similar results were observed when coupling the above oligoalkylamines with C12 120. The specific targeting mechanism is not mentioned in the above articles, but we infer that the liver-targeting may relate to the first pass effect of liver with intravenous injection or the protein corona formed on the vectors' surfaces. Beyond liver, researchers are exploring delivery systems for other organs of interest to fully realize the potential of RNA-based drugs.

#### Targeting of the lung and spleen

**Figure 6:**
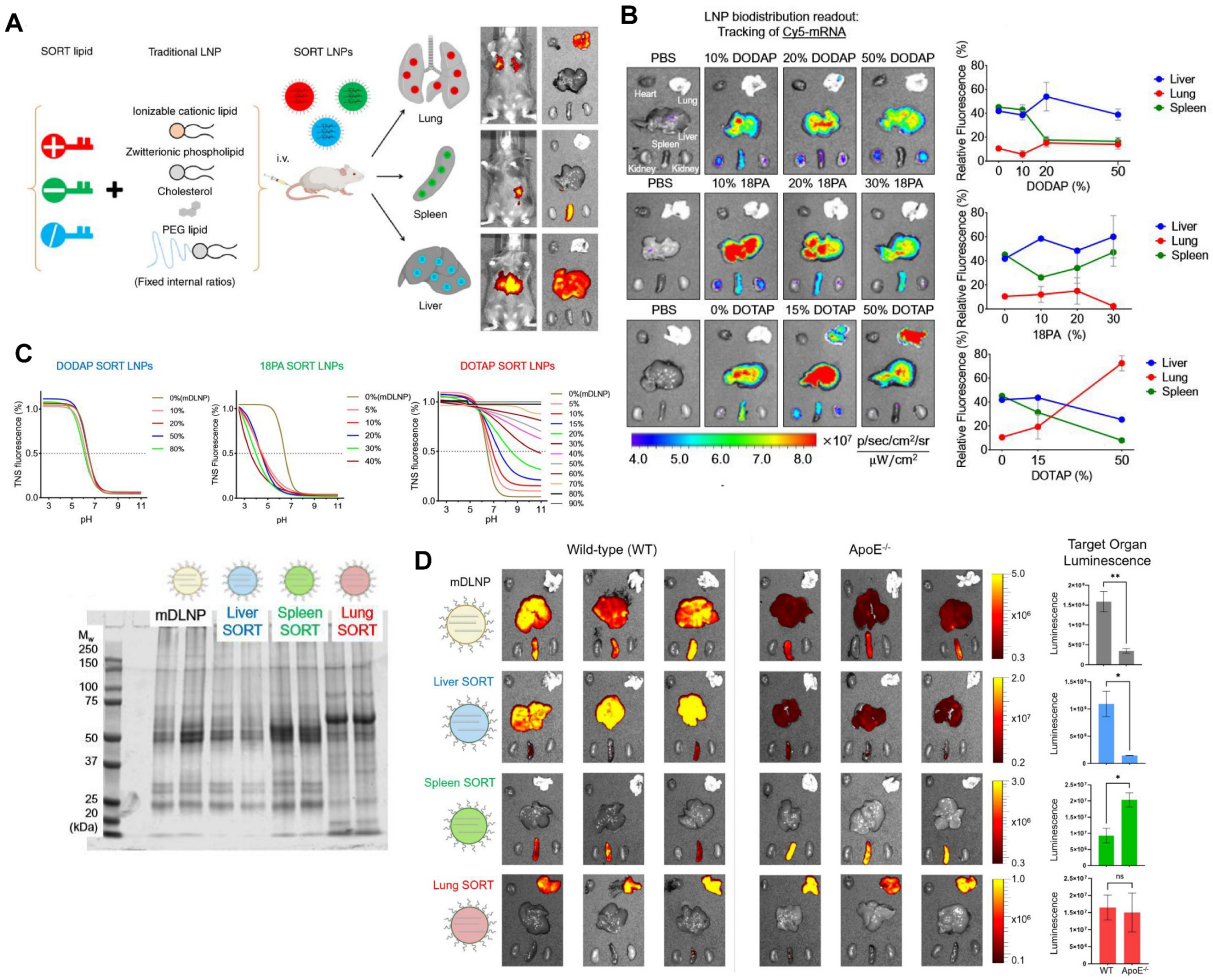
SORT molecules allowed LNPs to achieve targeted delivery of mRNA for different organs. (A) Addition of different SORT molecules mediated the tissue-specific targeting delivery of LNPs. Adapted with permission from 17, copyright 2020, Springer Nature. (B) Increase of percentage of different SORT molecules altered the bio-distribution of fluorescence of Cy5-mRNA. (C) The addition of different percentages of SORT molecules affected the p*K*_a_ of LNPs determined by TNS assay and plasma proteins absorbed in surface of LNPs visualized by SDS-PAGE. (D) The bioluminescence of functional proteins showed that the targeting of SORT LNPs in lung and spleen was ApoE-independent. Adapted with permission from 121, copyright 2021, National Academy of Sciences.

Typical LNPs are composed of four compositions and exert the characteristics of liver-targeting. Recently, Cheng *et al.* discovered that the presence of the fifth component, called 'Selective Organ Targeting (SORT)' molecule, could alter the organ-targeting effect of LNPs *in vivo*. The organ selectivity depended on the type and amount of SORT lipid added (Figure 6A-B). Notably, LNPs with four components primarily target the liver, but the luminescence activity would transfer from the liver to the spleen, and finally to the lung tissues with the increase of the fifth component-permanent cationic lipids, such as DOTAP, DDAB, and EPC. Encouragingly, the addition of negatively charged lipid, 18PA, led to spleen targeting. 14PA and 18BMP addition results demonstrated that the targeting ability was independent of the structure of negatively charged lipids used. In addition, ionizable cationic SORT lipids, such as DODAP and C12-200, enabled enhanced liver delivery without altering the tissue tropism 17,121. Further mechanism was investigated for this organ-specific targeting. SORT lipid molecules were shown to affect the apparent p*K*_a_ of LNP and the interaction between LNP and serum proteins (Figure 6C). Furthermore, targeting of LNPs to the spleen and lungs is apolipoprotein independent (Figure 6D) 121. It is worth mentioning that this targeting strategy has been verified to deliver Cas9/sgRNA ribonucleoprotein complexes (RNPs) and achieved gene editing successively in liver and lung of the mice model 122. These findings demonstrate tremendous potential, particularly in the field of gene therapy. Thus far, the LNP-SORT platform has been established and has the potential to be used in treating primary ciliary dyskinesia (PCD) and cystic fibrosis (CF) in the future 17.

**Figure 7:**
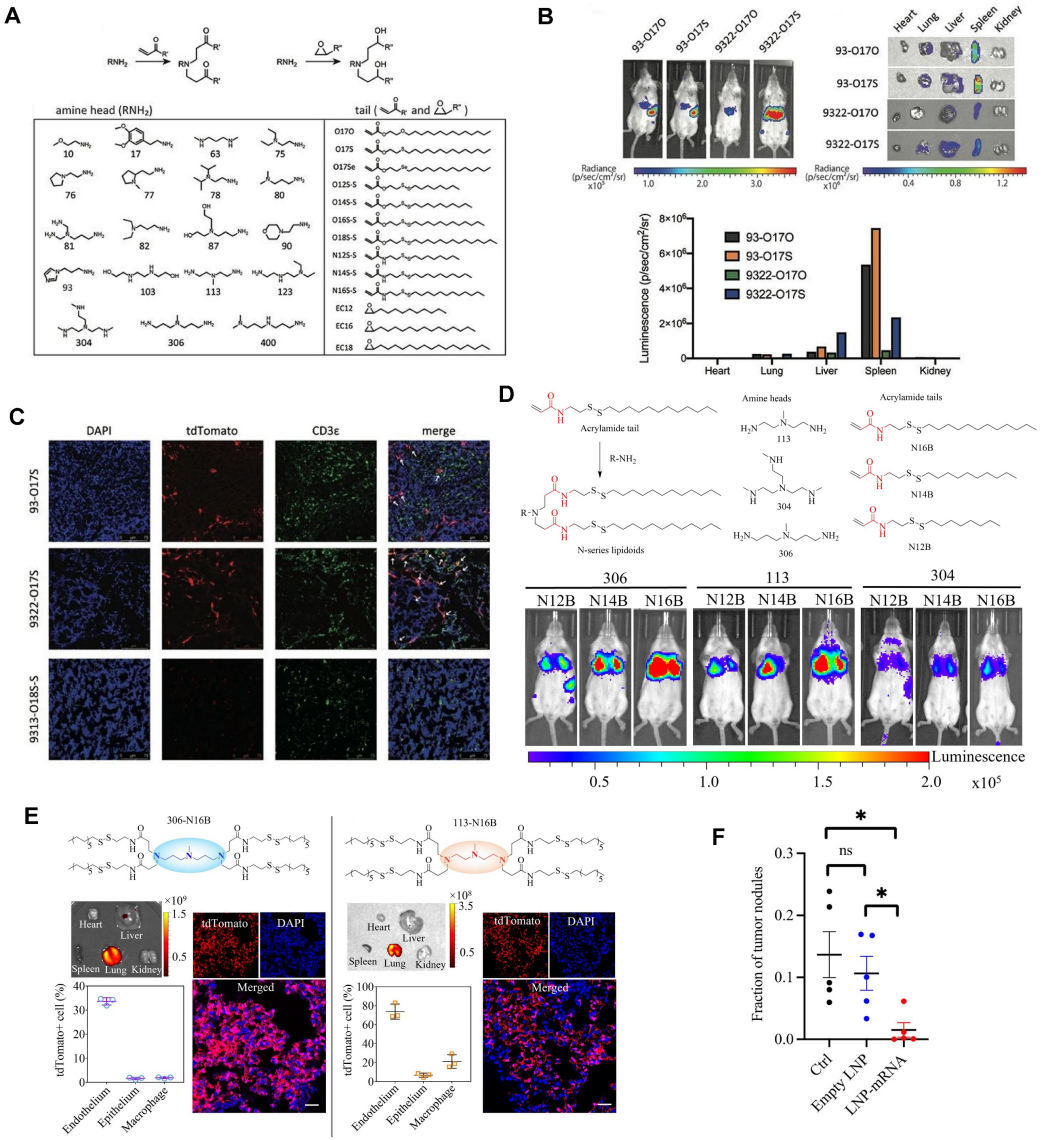
Imidazole-based lipidoids for spleen-targeting and N-series LNPs for lung-targeting. (A) Synthesis of imidazole-based lipidoids. (B) Screening LNPs according to bioluminescence images of whole body and each organ with IVIS. (C) Detection of tdTomato expression in spleen by confocal microscopy and T cells were marked by CDε antibody. Adapted with permission from 123, copyright 2020, Wiley-VCH. (D) Synthesis and screening of N-series LNPs by whole body bioluminescence images with IVIS imaging system. (E) Changing the head structure of N-series LNPs could target different pulmonary cell types represented by immunofluorescence images of lung tissue and quantification of tdTomato^+^ cell percentages in different pulmonary cell types of 306-N16B and 113-N16B. (F) Tsc2 mRNA-loaded LNP could exert therapeutic effect of inhibiting growth of tumor in lung. Adapted with permission from 115, copyright 2022, National Academy of Sciences.

Cationic lipid has been regarded as the critical component in LNPs, and its chemical tailoring provides another option for mediating organ-targeting. For instance, Xu and colleagues developed imidazole derived cationic lipids that could preferentially deliver mRNA to the primary T lymphocytes of the spleen, achieving cellular level targeting (Figure 7A-C). Subsequently, N-series LNPs with amide bonds in the lipidoid tails, particularly 306-N16B LNPs, were identified as the ideal candidates for lung targeting (Figure 7D). More importantly, altering the head group of N-series cationic lipid targeted different subcellular populations of lung as shown in Figure 7E 114,115,123. Following these, the lung targeting capacity of N-series LNPs was validated by delivering Tsc2 mRNA for the treatment of pulmonary lymphatic leiomyoma in a preclinical model (Figure 7F) 115. Therefore, tailoring the chemical structures of lipids, such as the amine head and linker, might provide a way forward for organ tropisms.

**Figure 8:**
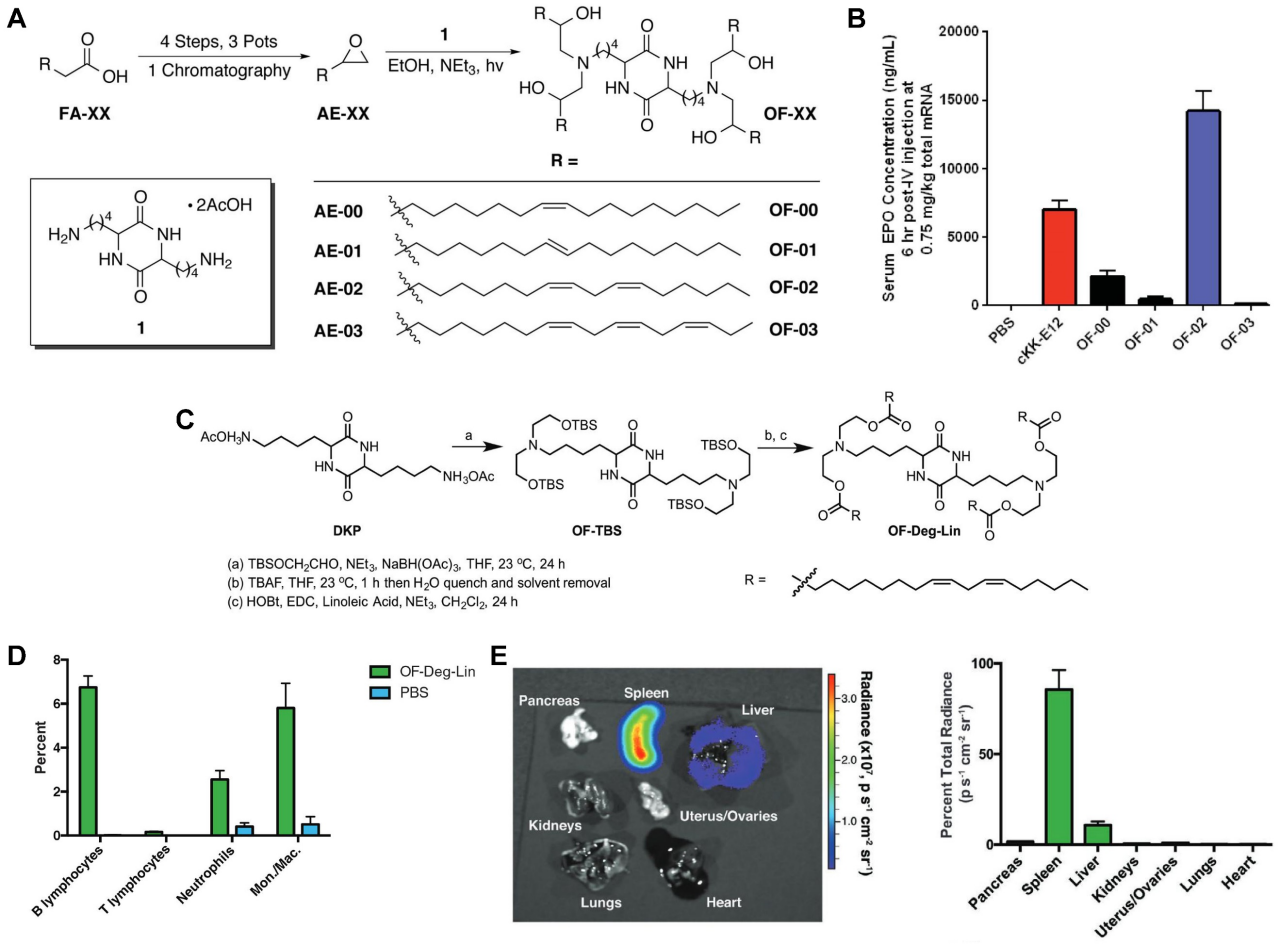
OF-XX series lipids for spleen-targeting. (A) Synthesis route of OF-XX lipids by ring opening reaction between alkenyl epoxides and polyamine core. (B) EPO expression following mRNA delivery with OF-XX LNPs and cKK-E12 LNP *in vivo*. Adapted with permission from 125, copyright 2016, Wiley-VCH. (C) Synthesis route of OF-Deg-Lin. (D) Quantification of cell populations labeled by Cy5 mRNA delivered with OF-Deg-Lin showed that B lymphocytes were the main targeted cell population. (E) Luciferase expression showed that OF-Deg-Lin LNPs could deliver FLuc mRNA to the spleen of mice. Adapted with permission from 126, copyright 2017, Wiley-VCH.

The spleen, which is rich in lymphocytes and macrophages, is an important immune organ. A library of ionizable amino-polyesters were synthesized *via* ring-opening polymerization of lactones and tertiary amino-alcohols. These polymers were incorporated in LNPs (APE-LNPs), which achieved mRNA expression in hepatocytes, pulmonary endothelial cells, and antigen presenting cells of the spleen 124. In another study, Anderson and colleagues synthesized a series of alkenyl amino alcohols (AAA) lipids through ring-opening reaction of alkenyl epoxides and a polyamine core as shown in Figure 8A. OF-02 LNP was identified as the top vector to achieve liver-targeting *in vivo*, showing superior delivery efficiency compared to cKK-E12 LNPs (Figure 8B) 125. Afterwards, based on OF-02 lipid, OF-Deg-Lin lipid was designed with a diketopiperazine core and four esterifiable unsaturated tails (Figure 8C). Further investigation showed that OF-Deg-Lin LNPs could target B lymphocytes of the spleen, inducing luciferase expression in the spleen after FLuc mRNA delivery (Figure 8D-E) 126. Additionally, OF-Deg-Lin was further modified *via* changing the carbon linker length from two to four to obtain OF-C4-Deg-Lin lipid, showing higher efficacy for mRNA delivery 127. Gomi *et al.* prepared a series of LNPs with alcohol-soluble phosphatidylserine (PS) molecules, which targeted spleen for mRNA delivery and translation, demonstrating application potential in immunotherapy and vaccines 128. Based on a siRNA vector TNT-a_10_, Dong and colleagues optimized the position of functional groups to obtain the TNT-b_10_. Further optimizing the formulation of TNT-b_10_ LLNs resulted in a high signal of mRNA expression in the spleen, which was 10 times higher than that in the liver with intravenous injection 129. Based on LNPs, Cao *et al.* combined helper-PBAEs and DOTAP to develop five-element nanoparticles (FNPs) for lung-targeted delivery of mRNA with high stability after lyophilization 130. Tombácz *et al.* combined the CD4 antibody with LNPs, achieving much higher accumulation of radiolabeled mRNA in the spleen compared to non-modified LNPs after systemic administration. Further expression of a reporter gene showed CD4-targeted LNPs could specifically deliver Cre mRNA to the spleen and lymph nodes 131. Notably, lymph nodes are place where various immune cells gather and immune responses occur and this research reminds that by conjugating antibodies or ligands for specific immune cells subsets, it is possible to achieve lymph node targeting for activating immunoreaction in some tumor or infection models.

**Figure 9:**
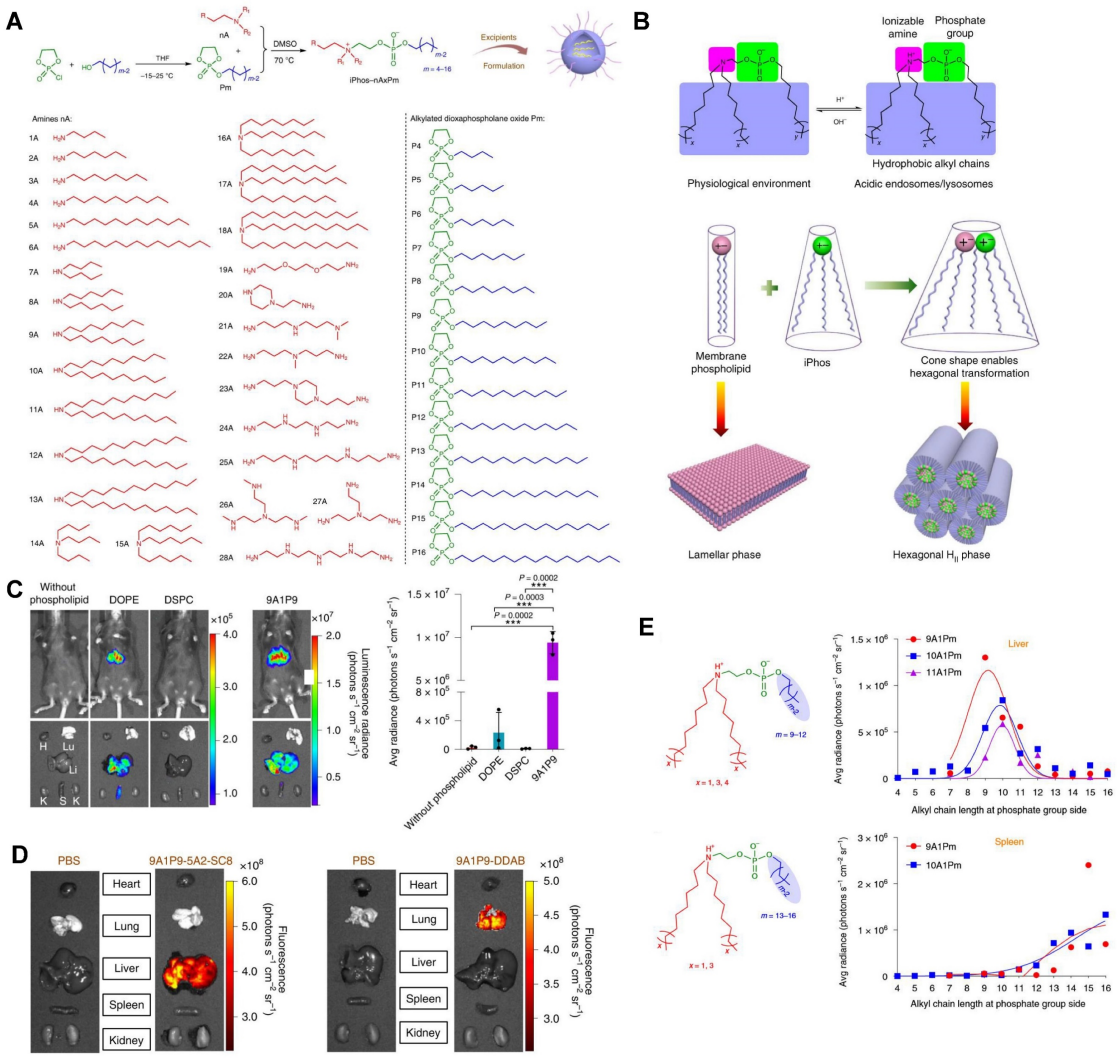
A series of novel iPhos for mRNA targeted delivery. (A) Synthesis routes of iPhos lipids by conjugating amines and alkylated dioxaphospholane oxide molecules. (B) Schematic illustration of hexagonal transition of biofilm phospholipids with the addition of iPhos lipids, which contained one zwitterionic head and three hydrophobic alkyl tails, in acidic environment. (C) Bioluminescence images and quantification of luciferase expression showed that the delivery efficiency of mRNA by iPhos 9A1P9 were superior to that of commonly used phospholipids, DOPE and DSPC. (D) Cas9 mRNA delivery and gene editing were achieved in liver and lung with 9A1P9-5A2-SC8 iPLNPs and 9A1P9-DDAB iPLNPs respectively. (E) iPhos with alkyl group length of 9 to 12 carbons resulted in highest mRNA expression in liver, 13 to 16 carbons resulted in highest mRNA expression in spleen. Adapted with permission from 31, copyright 2021, Springer Nature.

CRISPR/Cas9 gene editing technology has shown revolutionary impact on the gene therapy field. However, delivering the editing tool safely, effectively, and accurately to the target sites remains a significant challenge 27,28. To address this issue, it is necessary to improve the endosomal escape of the drug-loading nanoparticles and achieve extrahepatic targeting. A library of novel ionizable phospholipids (iPhos) with strong endosomal escape properties were designed and synthesized by Liu *et al.* (Figure 9A) 31. Most biofilm phospholipids adopted a bilayer architecture, but when iPhos entered the acidic endosomes, the tertiary amines of iPhos lipids would protonate to form a zwitterionic head and insert into endosomal membranes to produce a cone shape. This process would generate hexagonal transition and cause rupture of endosomes to release the contents (Figure 9B). iPhos based LNPs (called iPLNPs) were utilized to deliver mRNA or Cas9 mRNA/sgRNA in follow-up studies and this new delivery system achieved extremely high mRNA delivery superior to benchmark phospholipids, DOPE and DSPC (Figure 9C). Also, efficient CRISPR/Cas9 gene editing in liver and extrahepatic organs were achieved (Figure 9D). Moreover, the relationship between iPhos structures and organ selectivity were revealed: 1) The alkyl length next to the phosphate group could determine the organ selectivity: a shorter length of 9 to 12 carbons would deliver mRNA to the liver for protein expression, while a longer length (13 to 16 carbons) benefited spleen delivery (Figure 9E). 2) Selective mRNA expression or CRISPR/Cas9 gene editing could be achieved in the spleen, liver, and lungs with the delivery of 9A1P9 iPLNPs containing zwitterionic, ionizable cationic, and permanent cationic helper lipids, respectively 31. In another study, Miller *et al*. reported that zwitterionic amino lipids (ZALs) could co-deliver Cas9 mRNA and sgRNAs with a high efficacy *in vitro* and *in vivo*. Specifically, ZA3-Ep10 LNPs enabled gene editing in the liver, kidney, and lungs 32. High-throughput screening technique, as one of the effective methods to screen nanoparticles with extrahepatic tropism, is capable of quantifying numerous delivery nanoparticles and protein expression *in vivo* in a short time 132,133. Dahlman and colleagues developed a high-throughput screening technique, called FIND, which could significantly improve the screening efficiency of LNPs. Using this technique, they verified two LNPs, 7C2 and 7C3, which were synthesized based on the 7C1 and composed of different four compositions and ratios, could deliver Cas9 mRNA and sgRNA into endothelial cells of the spleen 133.

Researchers have also investigated the relationship between other vector structures and their targeting abilities, beyond LNPs. Kaczmarek *et al.* reported that PBAE DD90-C12-122 could deliver mRNA to the lungs after intravenous administration, and PEGylation increased the delivery efficacy. Further optimizations, such as varying the carbon chain lengths of alkylamine and the molar ratios of diacrylate and amines, would lead to polymeric nanoparticles with greater delivery potential targeting different subtypes of lung. Analysis of the lung cell types after administrating the optimized formulation of polymers showed the transfected cells were mainly among pulmonary endothelial cells and few immune cells 69,134. Recently, Kaczmarek *et al.* synthesized a library of PBAEs and identified two polymers, D90-C12-103 and DD90-C12-103, which used diacrylate-amine as the backbone, could deliver pDNA and mRNA to the lung of mice post systemic administration. Results showed that the fluorescence peak of mRNA was much higher than that of pDNA *in vivo* after delivery and area analysis of radiance flux and luminescent images at different time points demonstrated that DD90-C12-103 vector was superior to D90-C12-103 for mRNA delivery 135. A degradable polyester library modified with amino thiols and alkyl thiols was built and screened *in vitro* and *in vivo*. A top polymeric vector, PE4K-A17-0.33C12, formulated with 5% F127 and FLuc mRNA was verified to show high luminescence activity in the lung after intravenous administration 136. Recently, a polymersome library consisting of cationic and helper polymers was designed and synthesized by modifying poly(ethylene glycol) block poly(lactide-co-glycolide) (PEG-PLGA) with various oligopeptides and charged groups. PA9-ZP3 and PH9-Aln were identified which could achieve outstanding liver- and spleen-targeting respectively. Further investigation demonstrated the bisphosphonate group and oligo-histidine played an important role in spleen-targeting and the organ-selective delivery was relevant to the sizes, charges, protein corona formed on the surface of vectors 137. In summary, organ-targeting is largely associated with vector structures. Cationic materials with excess positive charges (as the main or helper component) trend to deliver RNA cargoes to the lung, while negatively charged component incorporation shows spleen tropism.

#### Targeting of other organs or tissues

To date, the liver, lung, and spleen are commonly targeted organs in current research, however, targeting other organs such as the eyes, skin, heart, and brain holds great potential but remains more challenging in clinical applications.

Despite the translation potential, most LNPs tend to accumulate in the liver, and overcoming the natural liver targeting characteristics of LNPs has become a key challenge in nano-vector research. Recently, Sahay group developed LNPs decorated with an oligomer peptide to deliver mRNA to neural retina in rodents and non-human primates after intravitreal administration. This breakthrough demonstrates the potential of LNP-mRNA in the treatment of inherited retinal diseases and represents significant progress in LNP penetrating biological barriers to achieve extrahepatic targeting 138. Xue *et al.* synthesized a series of LNPs with bisphosphonate (BP)-lipid, which showed higher affinity for bone-related fragments* in vitro* and higher bone-targeting *in vivo* compared to LNPs without BP-lipid 139. Another example of extrahepatic targeting involves decorating LNPs with CD5 antibody to produce CAR T cells transiently by delivering mRNA to target T cells. In a mouse model of heart disease, the targeted-LNPs encapsulating modified-mRNA could reduce the fibrosis degree of heart and restore heart function 140.

The skin is the largest organ in the human body and skin aging is related closely to the fibroblast in dermis. Recently, Francisco and Ferreira designed hundreds of polymers and selected six polymers with similar chemical structures composed of diacrylate, special amine, and bisacrylamide, which could transfect mouse fibroblasts with high efficiency *in vitro*. Among them, P1E28 containing alkyl alcohol side chain and piperazine rings with two tertiary amines showed the highest transfected efficiency. *In vivo* results showed that P1E28 polymeric nanoparticles delivered Cre mRNA to skin dermal fibroblasts with a higher delivery efficiency than endothelial cells, keratinocytes and macrophages. Further mechanism analysis indicated the ability of P1E28 to target skin fibroblasts might be mediated by CD26 and FAP overexpressed in fibroblasts 141.

Brain represents another hard-to-target organ, and the presence of BBB limits the therapeutic effect of brain diseases 97,142. One innovative solution is the use of neurotransmitter-derived lipidoids (NT-lipidoids), which can efficiently deliver drugs to the brain *via* intravenous injection. By lipidating the neurotransmitter, the formulated lipid nanoparticles could pass through the BBB by mediating receptors and enter the central nervous system (CNS) to eventually release encapsulated small molecule drugs, macromolecules and gene editing proteins in neuronal cells. It has been demonstrated that the addition of NT1-lipidoids to facilitate vectors penetrating the BBB is suitable for various types of LNPs 143. Above study reminds us that delivering the drugs through receptors in the brain is an effective strategy, and transferrin receptor (TfR) has been regarded as one of the most promising targets 142. Rodrigues *et al.* modified liposomes with cell-penetrating peptides and transferrin ligands, achieving the targeting of brain capillary endothelial cells expressing TfR 144. TfR has also been identified as a promising target for glioblastoma, a lethal brain cancer, as its expression is up to 100 times higher in cancer cells than that in healthy cells 145,146. Exosomes functionalized by T7 peptide with binding ability to TfR could deliver antisense miRNA oligonucleotides against overexpressed miR-21 in glioblastoma to reduce the tumor size *in vivo* 147. Additionally, nanoparticles coated with polysorbate 80 (PS 80) were developed to combine with apolipoprotein for interacting with lipoprotein receptors. By adjusting coating density, siRNA delivery across BBB was achieved for the treatment of brain diseases *via* inhibiting the expression of tau protein in a traumatic brain injury mouse model 148. Overall, these studies demonstrate that targeting BBB relevant receptors can be an effective strategy for delivering drugs into the brain.

#### Targeting of the tumor cells and immune cells

Cancer is one of the leading causes of death in humans. Targeting drugs provide a precise way to target tumor cells without affecting normal cells, reducing side effects and improving overall survival rates compared to traditional chemotherapeutic drugs 149. Currently, one of the main methods to achieve tumor targeting is by attaching antibodies or ligands (such as peptide ligands mentioned above 147) to vectors that bind to surface receptors of tumor cells 150. Monoclonal antibodies (mAbs) are considered to be the most promising candidates against cancer 151. Generally, mAbs have the function of both targeting and anti-tumor effect. Great progress has been made for siRNA delivery with the help of mAbs, but there is no successful clinical translation until now, which is limited by the acquirement of LNP optimization according to different mAbs to some extent 151. To address this limitation, Peer* et al.* developed a modular targeting platform called anchored secondary scFv enabling targeting (ASSET), which was a membrane-anchored lipoprotein integrated with LNP and could interact with Fc constant domain of the antibody. The results showed that siRNA-loaded LNPs formed by adding ASSET and RIg could be taken up by targeted cells *in vitro* and produce desired gene knockdown *in vivo*. Further research found that altering RIg could achieve targeting of various leukocyte subsets specifically, demonstrating the potential applications of this platform in different disease models 152. In another study, Peer *et al.* utilized the ASSET strategy to connect anti-Ly6c^+^ mAbs to LNPs, achieving targeted delivery of therapeutic mRNA to Ly6c^+^ inflammatory leukocytes of mice with inflammatory bowel disease. These results highlight the potential of ASSET in the field of cancers, inflammatory diseases, and rare genetic disorders 153.

Cancer immunotherapy is a highly effective approach to fight against cancer, including using cancer vaccines. However, a major obstacle lies in how to target dendritic cells (DCs) to activate the immune response. In a recent study, RNA-lipoplexes (RNA-LPX) without ligand decoration were designed to target DCs for the delivery of cancer antigens and activation of immunoreaction post intravenous administration. Through adjusting the charge ratio of formulation, the positively charged RNA-LPX primarily targeted the lungs and the fluorescence expression shifted from lung to spleen with a reduction of the cationic lipid content. Notably, in a phase I clinical trial (NCT02410733), three melanoma patients were treated with RNA-LPX encoding the tumor antigens, and the produced IFNα and T-cell responses demonstrated the broad applicability of the RNA-LPX considering that antigens of any tumor cells could be encoded by RNA 153. RNA-based immunotherapy is making rapid progress in cancer treatment, with the help of advanced delivery technology.

### Administration routes

**Figure 10:**
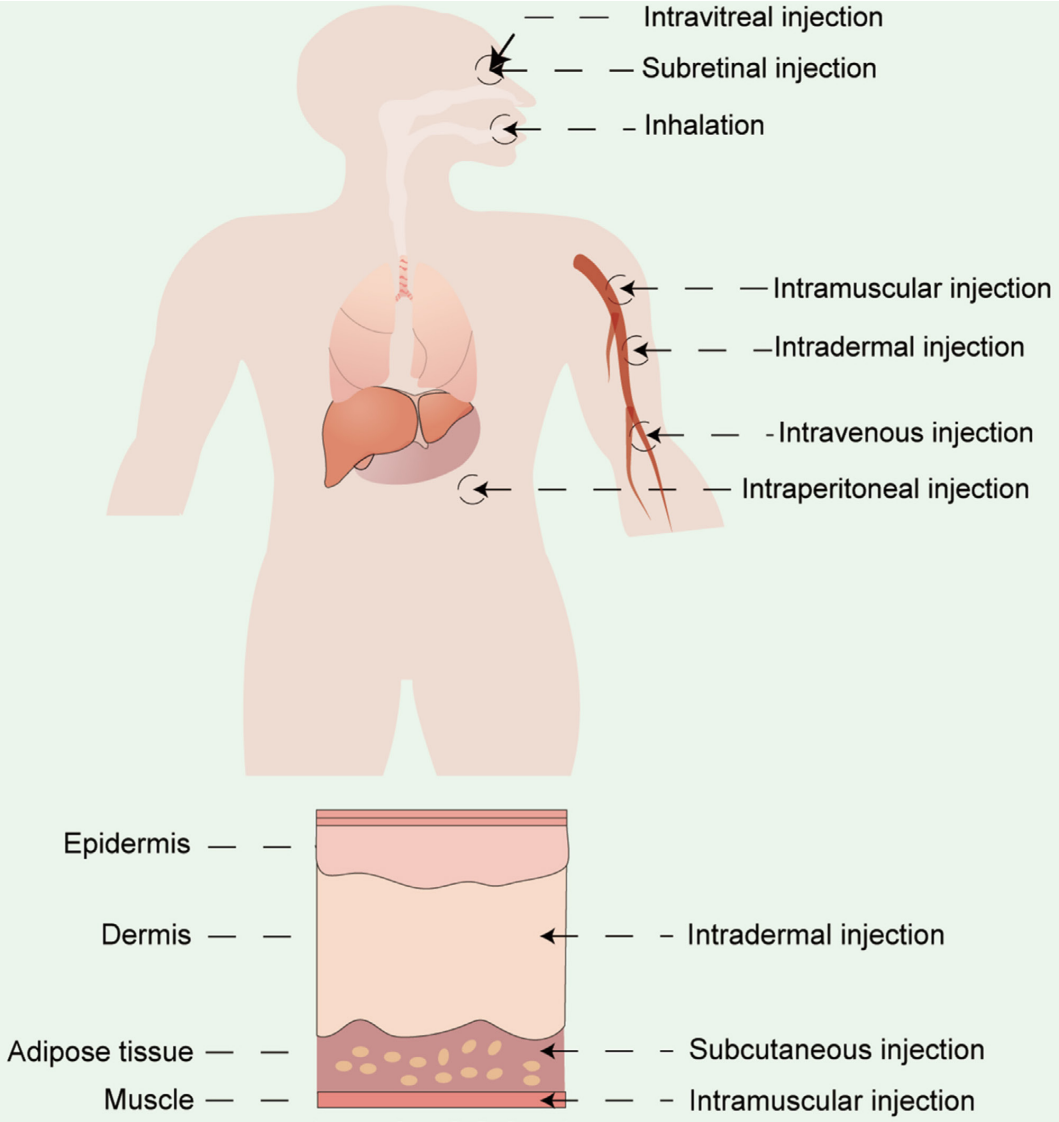
Different administration routes for RNA drugs, including systemic administration and local administration.

Apart from vector materials, the routes and sites of administration significantly influence the therapeutic outcomes and biodistribution of RNA drugs 154. Currently, the two primary modes of drug delivery in clinical practice include systemic and local administration. RNA drugs are usually administrated systemically *via* intravenous (i.v.) injection or locally administrated *via* subcutaneous (s.c.) injection, intramuscular (i.m.) injection, intradermal (i.d.) injections, and so on 155,156. Drugs can also be delivered by inhalation for pulmonary disease treatment 86,157 or by site-specific administration (e.g. heart 158, eyes 159,160, and brain 161) (Figure 10). The selection of the appropriate administration routes depends on the physicochemical properties and the desired therapeutic effects.

#### Systemic administration

Intravenous injection is commonly used for LNP-mediated delivery of mRNA or siRNA drugs. This route of administration allows the drugs to reach the target site through circulation. Notably, most LNPs administrated *via* the intravenous injection are prone to concentrate selectively in the liver because of its abundance of ApoE, exerting an essential influence on LNP-mediated RNA drugs 162. So far, systemic administration of non-viral vectors often results in significant hepatic specificity. Encouragingly, Melamed *et al.* reported that intraperitoneal administration of LNPs enabled a shift in specific mRNA expression from the liver to the pancreas 163. However, non-liver targeting through systemic administration to broaden the application prospect of RNA drugs still remains challenging 18.

Numerous efforts have been made to systemically deliver RNA to the lung for therapeutic use in pulmonary diseases. For instance, by rationally designing the structure of cationic lipids, Qiu *et al.* converted LNPs from liver-targeting to lung-targeting 115. In addition, suitable vectors enabled mRNA to be expressed in different cell subtypes of the spleen, including endothelial cells, macrophages, and antigen-presenting cells, enhancing the immune response 154,164,165. To date, the majority of RNA-based vaccines have depended on draining lymph nodes to reach specific sites primarily through intramuscular or subcutaneous injections 166. However, intravenous delivery of RNA might become another alternative approach, since there are plenty of non-immune cells at the injection sites. Luozhong *et al.* developed phosphatidylserine LNPs that could mediate effective mRNA expression in both lymph nodes and spleen after intravenous injection 167. In a transformative study, Cheng *et al.* reported a SORT strategy, which achieved the redirection of LNPs to extrahepatic organs by supplementing SORT molecules. Specifically, LNPs were shown to target the spleen by adding anionic lipids and modulating their formulations 17. Beyond LNPs, Liu *et al.* showed that zwitterionic phospholipidated polymers efficiently delivered mRNA to the spleen, and these polymers with specific amphiphilic and zwitterionic structures exhibited lymph node transfection capability 88. These findings demonstrate that designing appropriate delivery materials can enable RNA drugs to be delivered into immune organs through systemic administration, opening new opportunities for immunotherapy.

#### Local administration

As systemic delivery targets specific organs with intricate difficulties, local administration can be a preferable alternative for certain clinical applications. RNA-based drugs administered by topical delivery are expected to show a therapeutic effect at the specific site. In the case of treating pulmonary diseases, RNA drugs can be administrated to the lung *via* intravenous administration, inhalation or intratracheal injection 68,168,169. mRNA delivery by nebulization is shown to deliver more therapeutics to the lungs compared to systemic route 170. Efficient delivery of therapeutic RNAs is also an appealing strategy for ophthalmic applications requiring pro-chromatic expression of proteins in the retina. However, due to the complicated biological barriers of the ocular surface and the unstable physicochemical characteristics of RNAs, topical administration becomes the preferred administration method for ophthalmic treatment regimens. Devoldere and colleagues demonstrated that higher therapeutic effect of chemically modified mRNAs could be achieved by administrating on the photoreceptor side rather than through the vitreous side 171. Notably, a recent study reported an intradermal delivery of mRNA-loaded extracellular vesicles *via* a microneedle-based system and indicated that this technique could be used as a collagen replacement therapy for the treatment of skin diseases and aging 172. Interestingly, local administration may also produce systemic therapeutic effects. For instance, therapeutics delivered subcutaneously are capable of entering the systemic circulation as well as lymphatic circulation at a slow release rate, providing a longer period of time for the receptors that are closely linked to mediated cellular uptake 173. Specifically, RNA-based vaccines used in clinical practice are usually administered intradermally or intramuscularly, which are associated with the site of presence of antigen-presenting cells. Nowadays, many mRNA vaccines are administrated *via* intramuscular injection, enabling the drugs to be concentrated in the draining lymph nodes and trigger a strong immune response from T cells 61.

### Physicochemical properties

After numerous studies presenting the challenges faced by vectors in delivering RNA, it is evident that the barriers preventing the selective accumulation of drugs may be closely associated with the physicochemical properties of designed vectors 4,81. For the purpose of achieving site-specific accumulation of RNA drugs, researchers have scrutinized various physical parameters of non-viral vectors, including protein corona, particle size, surface and vector charge, and particle shape, with the aim of optimizing the potential for targeted delivery of nanoparticles.

#### Protein corona

The formation of protein coronas is believed to be closely associated with the biodistribution of nanoparticles in the body. Upon intravenous injection, the nanoparticles come into contact with the serum, leading proteins to adsorb on their surface and form a distinctive protein corona layer 121. The presence of the protein corona may alter the properties of the nanoparticles, with profound influences for their tissue specificity. For instance, most LNPs exhibit hepatic specificity *via* intravenous administration, owing to the adsorption of ApoE by binding to the LDLR that are over-expressed on the hepatocytes 121. Additionally, it has been validated that the knockdown of ApoE lowered the liver targeting of nanomaterials by delivering mRNA into a mouse model lacking ApoE expression 121. Although the mechanism of endogenous targeting is still not fully explored, researchers have striven to investigate the rules. It is discovered that when the PEG-lipids desorb from LNPs, specific protein binding to LNPs is promoted. The desorption rate of PEG-lipids with longer hydrophobic chains is slower, which might not conducive to formulating appropriate protein coronas and achieving organ targeted delivery 121. In parallel, nano-vectors with different structures and properties may adsorb different proteins, resulting in protein coronas with distinct components and properties. The formation of protein corona at the interface will alter the physicochemical characteristics of nanoparticles, exhibiting a crucial impact on their biodistribution and endocytosis 162. It has been demonstrated that LNPs with different p*K*_a_ values can influence their organ targeting. More specifically, the preferred p*K*_a_ range for liver, spleen, and lung targeting is 6.2~6.5, 2~6, and 7~9, respectively 121,174,175. These theories have implications for guiding the design of other delivery systems as well.

#### Particle size

The particle size and the dispersion of delivery vectors play a crucial role in constructing target delivery systems that meet different clinical needs. In order to overcome multiple intracellular and extracellular delivery barriers, attention should be paid to how the vector size affects the bio-interface interactions. Vectors of different sizes present different organ or tissue tropism *in vivo*. For instance, previous studies have revealed that nanoparticles with a diameter of approximately 5 nm are rapidly cleared by the renal filtration process 176. Most delivery vectors with particle sizes of 50~150 nm are prone to exhibit selective accumulation in the liver, such as LNPs, owing to the structure of endothelial cells 81. Furthermore, particles with diameters over 200 nm may possess splenic targeting capability due to the size of inter-endothelial cells. The pulmonary epithelium offers a large area (over 100 m^2^), making it an appealing target for RNA therapeutics upon systemic drug delivery. To maximize the drugs' efficacy in the lungs, particles with diameters of less than 3 μm are generally preferred, as they optimize deposition in the alveoli 169. Also, previous studies have reported that the transport capacity of polymer vectors in the blood can be significantly altered by varying their sizes 177. Specifically, the authors compared minor size differences to the effect of PAMAM dendrimers and results showed that the higher molecular weight polymers were able to accumulate more intensively in the brain 178. Since brain-targeted delivery usually requires receptor-mediated or vector-mediated transport passing through the blood-brain barrier, it is assumed that nanocomplexes with ultra-small dimension are more likely to cross the blood-brain barrier 177. Thus, small polyplexes functionalized with ligands, such as synthetic peptides, can be designed for targeting. Size is a crucial factor in determining how the particles are taken up by the cells, and it can significantly influence therapeutic sites of drugs.

#### Surface and vector charges

Surface and vector charges of nanoparticles are significant physicochemical properties that can be engineered to enhance the site-specific accumulation of RNA vectors and affect their delivery profile 179. Surface charge plays an important role in cellular internalization of the nanoparticles and contributes to overcoming the biological barriers during the drug delivery process 162. When the vectors show neutral or negative charges, they may have less adsorption of serum proteins, leading to a prolonged circulation time. In contrast, positively charged vectors tend to present a higher cellular uptake rate. Since polymers with neutral charges are prone to aggregation and precipitation, researchers usually use positively charged polyplexes to enhance cellular uptake *via* the interaction with negatively charged cell membranes. However, high-density surface positive charges can be correlated with corresponding toxicity, thus limiting their application to some extent. In accordance with the desired therapeutic effect, researchers have developed amphiphilic polymers or ionizable lipid nanoparticles that exhibit outstanding endosomal escape efficiency than conventional permanent positive vectors. These new materials are generally neutral under the physiological condition so as to mitigate the toxicity 177,180. In addition, Cheng *et al.* adjusted the lipid charges in LNPs, mediating controlled p*K*_a_ and specific organ tropism of nano-systems 17. It was shown that spleen-targeted delivery was more likely to be achieved when negatively charged lipids were introduced into the vectors, whereas the introduction of positively charged lipids might facilitate the delivery of RNA to the lungs. Vectors with different p*K*_a_ may likewise exhibit a propensity to accumulate in different organ tissues, which is one of the pivotal factors in the design of vectors. Therefore, the rational design of non-viral vectors with desired charges can lead to satisfactory therapeutic effects for RNA-based drugs.

#### Other properties

Other characteristics, such as particle shape and biodegradability, should also be taken into consideration when designing and evaluating the delivery performance of vectors. Studies have demonstrated that the shape of the nano-vectors plays a crucial role in its delivery journey. For instance, disc-shaped nanoparticles interact more effectively with the vessel wall and exhibit higher specificity for endothelial cells compared to spherical ones 181,182. Moreover, the stiffness of nanoparticles has been implicated to affect their clearance in the blood. For instance, the increased clearance of cholesterol-modified liposomes in the spleen might be due to their increased stiffness 183. Moreover, DeSimone and colleagues emphasized the significance of the elastic properties of the vectors on the circulation time and biodistribution of the therapeutics 184. In summary, physicochemical properties are underestimated in designing delivery systems, and their exploration in depth would help development of next-generation RNA vectors.

## Clinical advances in targeted delivery of RNAs

Over 20 years have passed since the first RNA-based drug was approved by FDA, and now several RNA drugs have been approved for therapeutic applications in different diseases, including TTR-mediated amyloidosis, duchenne muscular dystrophy, hypercholesterolemia, etc. Additionally, hundreds of candidate drugs are currently under clinical studies. Here, we present a summary RNA-based drugs that have been applied in clinics (Table 1), including information on the types of vectors used, administration routes, the diseases treated and the related organs.

### Clinical advances of mRNA drugs

mRNA therapy has gained immense popularity since the outbreak of COVID-19. Although no mRNA drug for cancer treatment has been approved by the FDA, there are several mRNA-based therapies for cancer in various stages of clinical trials, which can be divided into two major categories roughly based on the encoded antigens 196. The first category is cancer vaccines that encode tumor-associated antigens, representing the major type of mRNA-based cancer therapy. mRNA-4157 from Moderna & Merck has made a great breakthrough for the treatment of melanoma in phase II clinical trial (NCT03897881) and solid tumor in phase I clinical trial (NCT03313778) 197. And recently, the FDA has granted a breakthrough therapy designation to mRNA-4157/V940 in combination with PD-1 antibody for adjuvant therapy for high-risk melanoma to prevent postoperative recurrence, which inspired the progress of mRNA therapy in the field of vaccine. In addition, the companies plan to conduct phase III studies on melanoma and expand the treatment range to other tumor types, including non-small cell lung cancer. The second category is immuno-oncological treatments that use mRNA drugs. For instance, mRNA-2752 is encapsulated in LNPs to encode OX40L, IL-23, and IL-36γ, which can promote cytokine release and activate T cells to kill tumor cells. It has shown the effect in slowing tumor growth of patients with intratumoral injection 187. Currently, mRNA-2752 is in phase I clinical trial for the treatment of solid tumors and lymphoma (NCT03739931). The first inhaled mRNA drug MRT5005 is also in I/II clinical trial (NCT03375047) and aims to treat CF by delivering mRNA encoding CFTR to pulmonary epithelial cells through nebulization. Additionally, in the field of gene editing covering gene knockout and gene insertion, mRNA showed good clinical results. NTLA-2001, utilizing LNP encapsulating Cas9 mRNA and a sgRNA to target TTR, achieved knockout of TTR gene and decreased the concentration of TTR protein in serum by 52% with a dose of 0.1 mg/kg and 87% with a dose of 0.3 mg/kg in clinical phase I (NCT04601051). Additionally, NTLA-2002, targeting *KLKB1* gene, could block production of kallikrein and further reduce bradykinin to treat hereditary angioedema. In clinical I/II trial (NCT05120830), the NTLA-2002 encapsulating Cas9 mRNA and *KLKB1-*specific sgRNA delivered by LNP resulted durable reductions of kallikrein protein in plasma with well tolerability.

All the mRNA drugs mentioned above utilize LNPs as the delivery vectors, highlighting their significance in the field of RNA-based drugs. However, one exception is AZD-8601 mRNA, which has entered clinical trial (NCT03370887) for heart failure. AZD-8601 mRNA is formulated in citrate buffered saline with biocompatibility and is delivered by epicardial injections to express vascular endothelial growth factor A protein directly. Developed by AstraZeneca and Moderna, this drug increased left ventricular ejection fraction and enhanced heart function compared to placebo group 188. Despite the limited mRNA drug research in the past, recent laboratory experiments and clinical trials have demonstrated the potential of mRNA drugs in various fields, including protein replacement therapy, cancer immunotherapy, and gene editing, covering the therapy of liver, lung, heart-related diseases, and tumors. Table 2 provides some examples of mRNA drugs in clinical trials for diseases in different organs and tissue sites.

### Clinical advances of other RNA drugs

Most of these drugs approved by FDA target the liver, particularly siRNA drugs. In addition to the five siRNA drugs receiving market authorization, there are also some siRNA drugs in clinical trials and most of them are designed to treat liver-related diseases, such as Nedosiran. Primary hyperoxaluria (PH) is a metabolic disease caused by abnormal liver metabolism, resulting in excessive oxalate production from glyoxylate *via* lactate dehydrogenase (LDH) and excretion of oxalate in urine, which can cause severe kidney injury or kidney failure 189-191. LDHA and LDHB are two subunits of LDH encoded by their respective mRNA. Researches reveal that conjugating RNA with *N-*acetylgalactosamine (GalNAc) can achieve liver-targeting and the mechanism is that the GalNAc can combine with asialoglycoprotein receptors (ASGPRs), which are overexpressed by hepatocytes specifically 189. Nedosiran is a double-stranded siRNA conjugated with GalNAc and can combine with mRNA encoding LDHA, reducing the generation of LDH proteins and thus inhibiting the production of oxalate in the liver 189. Nedosiran is currently in phase II clinical trial (NCT05001269), and previous laboratory studies and clinical trials showed that this siRNA drug could target liver specifically to knock out LDHA without affecting other organs like muscle in animal models. It can also significantly reduce the oxalic acid content in urine after a subcutaneous injection in patients with high safety and tolerability 189,192,193. Cemdisiran is another promising siRNA drug conjugated with GalNAc that targets the liver to treat immunoglobulin A nephropathy *via* inhibiting activation of complement component 5. Alnylam declared that in phase II clinical trials (NCT03841448), Cemdisiran reduced the ratio of urinary protein and creatinine (UPCR) by an average of 37% at 24 hour without adverse effects after subcutaneous administration compared to placebo group 194. In summary, the therapeutic applications of siRNA are wide, covering the fields of metabolic diseases, ocular diseases, infectious diseases, tumors, and so on 194.

Similar to siRNA, ASO drugs on the market are primarily used to treat patients with rare diseases, but they have a wider range of target organs, including liver, muscle, eyes, and so on. The diseases they address include nervous system diseases, muscle diseases, cardiovascular diseases, and metabolic diseases. In addition to rare diseases, an increasing number of ASO drugs are being developed for other common diseases and are currently in middle- or late-stage clinical trials 195. For instance, Pelacarsen, an ASO drug that targets mRNA expressing LPA in hepatocytes, reduced the level of lipoprotein(a) by 35-80% in phase II clinical trial (NCT03070782). This reduction is promising for patients with chronic kidney disease since elevated lipoprotein(a) levels are associated with kidney damage 196. CDR132L is a synthetic ASO that function as the inhibitor to target miR-132 in heart failure to normalize cardiomyocytes. Thum *et al.* have demonstrated the powerful effect of CDR132L in large pig animals and the results showed that this drug significantly improved the systolic and diastolic function of the heart and reversed cardiac remodeling after repeated monthly administration 197. Phase 1b clinical trial (NCT04045405) demonstrated the effectiveness in patients with heart failure after intravenous administration with safety and tolerability in the dose range of 0.32~10 mg/kg 198. The development of delivery strategies targeting other tissues instead of the liver is the key to expand the applied range of small RNA drugs, and pulmonary administration is a reliable way to achieve lung targeting. IONIS-ENAC-2.5Rx and QR-010 that have completed phase I or II clinical trials (NCT03647228, NCT02564354/NCT02532764) are two ASO drugs for the treatment of CF *via* aerosol administration 195. And results from clinical trials demonstrated the effectiveness of these ASO drugs in treating CF 194,195.

It is noteworthy that over half of the clinic trials on RNA-based drugs are focused on mRNA, highlighting the huge potential of mRNA therapeutics in RNA-based therapy at present. However, the majority of active clinic trials involving mRNA are derived from COVID-19 vaccines and for the indication of infections. If we exclude these vaccine-related trials, most remaining trials are still in the early-stage 185. This reminds us that more progress should be made to expand the field of mRNA therapy and diversify its applications in other diseases.

## Conclusion and Outlook

Since the discovery of the therapeutic potential of RNA drugs half a century ago, numerous delivery platforms have emerged to enable the safe and efficient delivery of RNA. Through the diligent efforts of researchers in library screening and rational design of delivery systems, several RNA-based drugs have received marketing approval or are currently undergoing clinical trials. As the focus has gradually shifted towards better adapting to clinical needs, efforts are being made to target specific sites for the treatment and reduce the side effects of RNA therapy. With a deeper understanding of the targeting mechanism, researchers are striving to achieve targeted therapies for RNA drugs by using different categories of vectors for delivery, tailoring vector chemical structures, adapting various administration routes, and altering physicochemical properties of vectors. These major advances in delivery systems provide great potential for RNA therapy.

Although the first siRNA drug utilizes the LNP vector, the following four siRNA approved drugs instead employ the GalNAc technology, which has become the primary strategy for siRNA-targeted delivery to hepatocytes. While in the field of mRNA-based therapy, advanced LNPs have been developed in two widely inoculated vaccines for COVID-19, known as BNT162b2 and mRNA-1273. This breakthrough has facilitated the exploitation of mRNA-target delivery systems, which have enabled selectivity for organs or tissues by modifying the lipid chemical structures and their formulations. Despite the progress, much work remains to be undertaken to achieve better clinical translation. 1) A deeper understanding of the targeting mechanism is required to develop universal guidelines for achieving targeting to each organ or even different cell subtypes, thus facilitating the design of next-generation delivery systems. 2) Additional investigations on the physicochemical properties, morphology, stability, and other characteristics of the vectors are required to facilitate further targeting and druggability. One can design vectors with specific sizes or charges to achieve targeting delivery of RNA according to the characteristics of different organs and tissues. 3) More attention can be paid to the inherent targeting ability of some natural vectors and figuring out the mechanism of their native tropism, which can guide the future specific targeting. Identifying the exact mechanism of targeting delivery will fundamentally help to design the appropriate vectors to achieve precise delivery. 4) Biocompatibility of vector materials should be considered to reduce toxicity and avoid undesired immunogenicity. 5) Inspired by protein corona-guided and GalNAc-conjugated targeting delivery system, more efforts should be made to screen specific receptors possessed by cell subsets for cellular level targeting. 6) Stepwise targeted drug delivery strategy from organ, tissue, cell to organelle may be utilized to overcome obstacles in delivery process and improve delivery precision.

Overall, the targeted RNA-based therapies have already demonstrated tremendous therapeutic potential in numerous clinical applications. As an integral part of RNA therapeutics, the development of a more precise delivery system is of undeniable significance. The prospects are promising that targeted RNA drugs have the potential of utility in diverse disease fields. Although some challenges remain for targeted RNA delivery, the enormous and continuous advances in RNA therapy will undoubtedly improve our health and lives.

## Figures and Tables

**Table 1:** The ribonucleic acid therapeutics approved by FDA.

Products	Category of ribonucleic acids	Approval year	Delivery vectors	Administration routes	Diseases	Targeted organs
Fomivirsen	ASO	1998 (withdrawn)	-	Intravitreal	Cytomegalovirus infection	Eye
Pegaptanib	Aptamer	2004 (withdrawn)	-	Intravitreal	Wet Macular Degeneration	Eye
Mipomersen	ASO	2013 (withdrawn)	-	Subcutaneous	Hypercholesterolemia	Liver
Eteplirsen	ASO	2016	-	Intrathecal	Duchenne muscular dystrophy	Skeletal muscle
Nusinersen	ASO	2016	-	Intrathecal	Spinal muscular atrophy	Spinal nerves
Inotersen	ASO	2018	-	Subcutaneous	TTR-mediated amyloidosis	Liver
Patisiran	siRNA	2018	LNP	Intravenous	TTR-mediated amyloidosis	Liver
Volanesoren	ASO	2019	-	Subcutaneous	Familial chylomicronemia syndrome	Liver
Golodirsen	ASO	2019 (confirmatory trial required)	-	Subcutaneous	Duchenne muscular dystrophy	Skeletal muscle
Givosiran	siRNA	2019	GalNAc	Subcutaneous	Acute hepatic porphyrias	Liver
Viltolarsen	ASO	2020	-	Intravenous	Duchenne muscular dystrophy	Skeletal muscle
Inclisiran	siRNA	2020	GalNAc	Subcutaneous	Hypercholesterolemia	Liver
Lumasiran	siRNA	2020	GalNAc	Subcutaneous	Primary hyperoxaluria type 1	Liver
Casimersen	ASO	2021	-	Subcutaneous	Duchenne muscular dystrophy	Skeletal muscle
BNT162b2	mRNA	2021	LNP	Intramuscular	COVID-19 (emergency use)	-
mRNA-1273	mRNA	2021	LNP	Intramuscular	COVID-19 (emergency use)	-
Vutrisiran	siRNA	2022	GalNAc	subcutaneous	TTR-mediated amyloidosis	Liver

**Table 2:** The clinical trials of mRNA targeting different tissues or organs.

Name	Delivery vectors	Administration routes	Diseases	Clinicaltrials.gov identifier	Phase
Liver					
ARCT-810	LNP	Intravenous	Ornithine transcarbamylase deficiency	NCT05526066	II
NTLA-2001	LNP	Intravenous	TTR-mediated amyloidosis with polyneuropathy	NCT04601051	I
mRNA-3745	LNP	Intravenous	Glycogen storage disease	NCT05095727	I
mRNA-3705	LNP	Intravenous	Isolated methylmalonic acidemia	NCT04899310	I/II
mRNA-3927	LNP	Intravenous	Propionic acidemia	NCT05130437	I/II
Lung					
ARCT-032	LNP	Inhalation	Cystic fibrosis	NCT05712538	I
MRT5005	LNP	Inhalation	Cystic fibrosis	NCT03375047	I/II
VX-522	LNP	Inhalation	Cystic fibrosis	NCT05668741	I
Heart					
AZD8601	No vector	Epicardial Injection	Heart failure	NCT03370887	II
mRNA-0184	Unknown	Intravenous	Heart failure	NCT05659264	I
Tumor					
mRNA-4157	LNP	Intramuscular	Melanoma	NCT03897881	II
mRNA-4157	LNP	Intramuscular	Solid tumor	NCT03313778	I
mRNA-2752	LNP	Intratumoral	Solid tumor Lymphoma	NCT03739931	I
BNT111	Liposome	Intravenous	Melanoma	NCT04526899	II
BNT122	Liposome	Intravenous	Colorectal cancer	NCT04486378	II
BNT151	Liposome	Intravenous	Solid tumor	NCT04455620	I/II
CV9201	Protamine	Intradermal	Non-small cell lung cancer	NCT00923312	I/II
CV-9202	Protamine	Intradermal	Non-small cell lung cancer	NCT03164772	I/II

## Abbreviations

mRNA: messenger RNA
siRNA: small interfering RNA
miRNA: microRNA
ASO: antisense oligonucleotide
UTRs: untranslated regions
IVT: *in vitro* transcribed
LNP: lipid nanoparticle
tRNA: transfer RNA
CRISPR: Clustered Regularly Interspaced Short Palindromic Repeat
Cas9: CRISPR-associated protein 9
sgRNA: single-guide RNA
RISC: RNA-induced silencing complex
TTR: transthyretin
AHP: acute hepatic porphyrias
TMTME: too many targets for miRNA effect
PEG: polyethylene glycol
LSECs: liver sinusoidal endothelial cells
PEI: polyethyleneimine
PBAE: poly(β-amino ester)
mPEG-CL: poly(ethylene glycol-block-caprolactone)
mPEG-LA: poly(ethylene glycol-block-lactide)
LPNs: lipid-polymer hybrid nanoparticles
GO: graphene oxide
EPR: enhanced permeability and retention
CFTR: cystic fibrosis transmembrane conductance regulator
CARTs: charge-altering releasable transporters
EVs: extracellular vesicles
BBB: brain-blood barrier
MSC: mesenchymal stem cell
INPs: inorganic nanoparticles
AuNPs: gold nanoparticles
SAR: structure-activity relationships
Pten: Phosphatase and tensin homolog
ApoE: apolipoprotein E
LDLR: low-density lipoprotein receptor
LLNs: lipid-like nanoparticles
HA: hemophilia A
SORT: Selective Organ Targeting
RNPs: ribonucleoprotein complexes
PCD: primary ciliary dyskinesia
CF: cystic fibrosis
AAA: alkenyl amino alcohols
PS: phosphatidylserine
FNPs: five-element nanoparticles
iPhos: ionizable phospholipids
ZALs: zwitterionic amino lipids
PEG-PLGA: poly(ethylene glycol) block poly(lactide-co-glycolide)
BP: bisphosphonate
NT: neurotransmitter
CNS: central nervous system
TfR: transferrin receptor
PS 80: polysorbate 80
mAbs: monoclonal antibodies
ASSET: anchored secondary scFv enabling targeting
DCs: dendritic cells
RNA-LPX: RNA-lipoplexes
PH: primary hyperoxaluria
LDH: lactate dehydrogenase
GalNAc: N-acetylgalactosamine
asialoglycoprotein receptors: ASGPRs
UPCR: urinary protein and creatinine
